# Graphene quantum dot based materials for sensing, bio-imaging and energy storage applications: a review

**DOI:** 10.1039/d0ra03938a

**Published:** 2020-06-23

**Authors:** Y. Ravi Kumar, Kalim Deshmukh, Kishor Kumar Sadasivuni, S. K. Khadheer Pasha

**Affiliations:** Department of Physics, VIT-AP University Amaravati Guntur-522501 Andhra Pradesh India khadheerbasha@gmail.com +91-9894665388; New Technologies – Research Center, University of West Bohemia, Univerzitní 8 30614 Plzeň Czech Republic; Center for Advanced Materials, Qatar University P.O. Box 2713 Doha Qatar

## Abstract

Graphene quantum dots (GQDs) are an attractive nanomaterial consisting of a monolayer or a few layers of graphene having excellent and unique properties. GQDs are endowed with the properties of both carbon dots (CDs) and graphene. This review addresses applications of GQD based materials in sensing, bioimaging and energy storage. In the first part of the review, different approaches of GQD synthesis such as top-down and bottom-up synthesis methods have been discussed. The prime focus of this review is on green synthesis methods that have also been applied to the synthesis of GQDs. The GQDs have been discussed thoroughly for all the aspects along with their potential applications in sensors, biomedicine, and energy storage systems. In particular, emphasis is given to popular applications such as electrochemical and photoluminescence (PL) sensors, electrochemiluminescence (ECL) sensors, humidity and gas sensors, bioimaging, lithium-ion (Li-ion) batteries, supercapacitors and dye-sensitized solar cells. Finally, the challenges and the future perspectives of GQDs in the aforementioned application fields have been discussed.

## Abstract 2

Graphene quantum dots (GQDs) are an attractive nanomaterial consisting of a monolayer or a few layers of graphene having excellent and unique properties.

## Graphical abstract

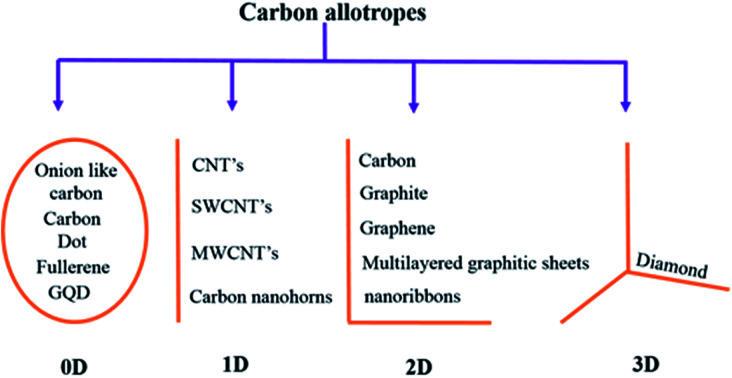

## 1. Introduction

**Fig. 1:**
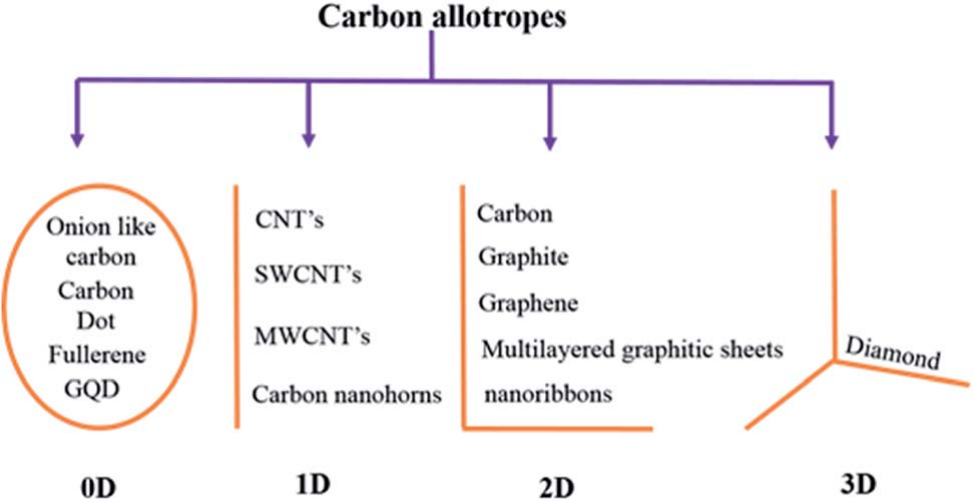
Classification of carbon materials.

Carbon is an astonishing material and is the most abundant material present in the form of coal, being one of the reasons for the survival of life in the world. It has amazed us once again recently in the form of graphene.^1^ The football-shaped fullerene, also an allotrope of carbon, was discovered in 1985 through small and needle-like carbon nanotubes (CNTs) which were first characterized fully in 1991.^2^ These recent discoveries of amazing allotropes of carbon have garnered great attraction and interest from scientists of all disciplines of science. The classification of carbon in graphitic forms includes zero-dimension (0D), one-dimension (1D), two-dimension (2D) and three-dimension (3D) graphite as shown in Fig. 1. 2D graphene is a single-layered nanomaterial peeled off from multi-layered graphite because of the van der Waals force among the layers. It is one atom thick carbon material which is the strongest and thinnest material ever measured in the Universe.^2,3^ Graphene was discovered in 2004 by a group of researchers from Manchester University, UK, and it was regarded as the miracle material of the 21^st^ century.^4–7^ The graphene and graphene-related series of materials include several similar nanostructures but different nomenclature which indicate that the carbon material contains a single or a few monolayers of graphene. Presently, different advanced methods are available for careful handling and manufacturing of graphene as well as its derivatives, which yield products with different sizes and content of debris such as C, O, H or surface groups such as carboxyl, carbonyl, epoxy and hydroxyl.^8–10^

Carbon dots (CDs) is often used as a general name for class of different carbon materials like carbon quantum dots (CQDs), graphene quantum dots (GQDs), carbon nanodots (CNDs) and carbonized polymer dots (CPDs). These forms of the CDs family can be classified based on the specific properties, surface groups and carbon core structures. The CQDs have crystal lattice with surface chemical groups and are spherical in shape, which possess quantum confinement effect (QCE) and intrinsic state luminescence. The photoluminescence wavelength offered by CQDs can be tuned by regulating the size of CQDs.^11^ Whereas, GQDs have obvious graphene lattice and comprise of single or few sheets of graphene fragments. The height of these GQDs is usually less than ten graphene sheets with a lateral dimension of less than ∼100 nm.^12^ In GQDs surface groups are attached either on the interlayer defects or on the edges which contribute to distinct QCE and edge properties.^13^ The QCE in GQDs not only comes from its size but is also governed by the conjugated π-domains, which are isolated on the graphene planes.^14^ On the other hand, CNDs usually don't exhibit polymer features and lattice structure but possess surface chemical groups and a high degree of carbonization. There is no role of the QCE in the photoluminescence properties and is mainly governed by the subdomain states and defect states present in the graphite carbon core.^15^ Another class is CPDs, which comprises carbon/polymer hybrid structure, where a considerable amount of polymer/functional groups are attached to the surface and carbon core. In CPDs molecular states, surface states, subdomain states and crosslink emission effect is mainly responsible for photoluminescence properties.^16^ A detailed review of the evaluation of carbon dots and classification based on the structure and properties is done by Xia *et al.*^16^

In the past decade, researchers have worked on the successful development of 0D GQDs in lines of the 2D graphene. GQDs consist of a monolayer or a few monolayers of graphene-related to quantum confinement and edge effects.^8^ These kind of materials offers several superior qualities like robust chemical inertness, fluorescence activity, photostability, tunable low cytotoxicity, luminescence emission and excellent biocompatibility, high solubility, high surface area, long term opposition to photobleaching, and better surface grafting.^17,18^ These properties, in turn, offer the opportunity to explore novel structural, optical and electrical phenomena that are unavailable in other materials. The adjustment of electron and quantum confinement behaviour of GQDs has become supremely attractive especially in comparison to graphene. These advanced properties make this material an encouraging candidate for several applications such as sensors, biosensors, bioimaging, photovoltaic and energy storage devices *etc.*^19,20^ GQDs exhibit excellent solubility in organic solvents such as dimethylformamide (DMF), tetrahydrofuran (THF), acetone, dimethyl sulfoxide (DMSO) and ethanol.^21^ However, the improved solubility in water-based solvents has profoundly influenced its application in the field of bioimaging and targeted drug delivery systems.^22^ The water solubility ability comes from the hydroxyl and carboxyl containing moieties attached at the edges of GQDs.^22^ As surface functionalities empower the hydrophilic nature of GQDs, it can be tuned by chemical methods and strongly depends on the synthesis method.

Despite several advantages and superior properties, the research on GQDs is quite at an initial stage, and many drawbacks of GQDs have yet to be overcome. Even though there are many significant advantages and potential applications evolved, additional exploration to boost the properties of the material is mandatory to overcome the limitations.^23^ Unlike graphene sheets, GQDs are 0D graphene segments that exhibit bandgap which is accountable for their unique optical and electrical properties. Due to small size, GQDs displays a quantum size effect. To exploit these unique properties and establish a wider application, several challenges have to be addressed. One such challenge is to ascertain a precise structure–property relationship. The chemical synthesis route of GQDs results in significant inhomogeneity in surface functionality and dimension. With such large variation in chemical functionality and size, it is difficult to analyze the mechanism of their unique properties. Moreover, the photoluminescence and quantum confinement properties of GQDs possess a strong size-dependent relationship. The large deviation in chemical structure and size comes from different synthesis process. Thus, in terms of fundamental and applied perspectives, it is important to realize the correlation between the synthesis process dependent size variation and optical properties. This review mainly provides a dedicated study in terms of the GQDs synthesis process, size-dependent photoluminescence properties and quantum size effect of GQDs. Further, the review illustrates the current improvement in the applications of bare and functionalized GQDs constructed due to their outstanding properties. The presently offered methods of GQDs functionalization and their features related to their dimension, nobbling, surface adaptation, and solvents have been explored in this review. Furthermore, the applications of bare and functionalized GQDs in sensor, energy storage and biological fields have been discussed. Finally, this review will provide an outlook and develop new knowledge of GQDs and their potential applications.

## 2. Synthesis of GQDs

**Fig. 2:**
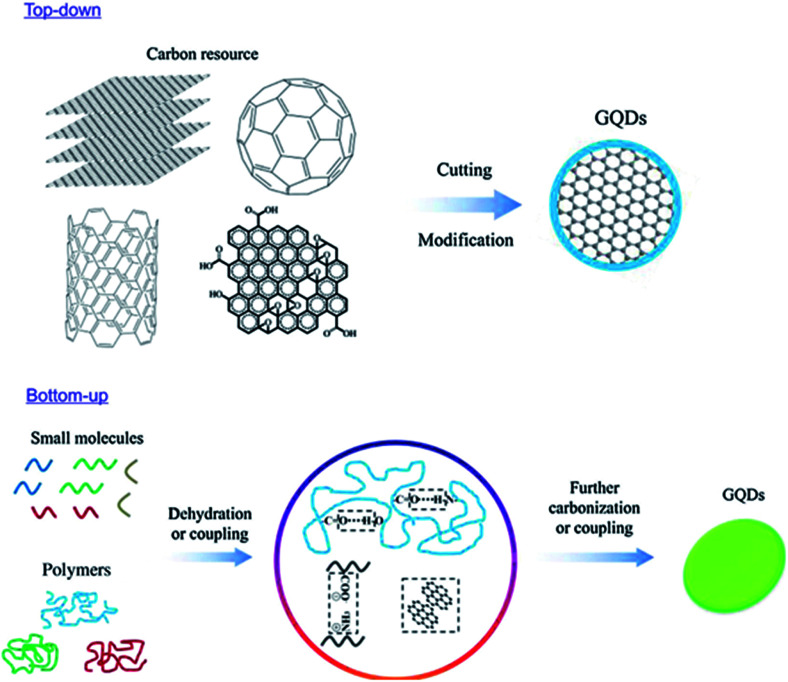
Two approaches to synthesize fluorescence GQDs: the “top-down” splitting from large molecules and “bottom-up” from small molecules. Reproduced with permission from ref. 25, Copyright 2018, De Gruyter.

Material synthesis is the major and foremost process before getting the material to the particular application. Generally, the variations in the result of applications are depended on the morphology and properties of the material which is mainly governed by the synthesis process. Hence, the researchers seriously need to concentrate and give attention to the process of material synthesis. GQDs are produced from carbon-rich materials such as fullerene, glucose, graphite, graphene oxide (GO), CNTs and carbon fibres (CFs) which are used as precursors. Two major methods for GQDs synthesis are followed, *i.e.* top-down and bottom-up approaches. Such kinds of techniques are complicated for the synthesis of the conventional semiconductor quantum dots.^24^ Later, carbonization or controllable synthesis methods were introduced to obtain the GQDs from appropriate organic molecules or polymers as shown in Fig. 2.^25^

The controllable synthesis is accurate but complex, requiring several processing steps to achieve GQDs having a large aspect ratio. While using the carbonization method, GQDs achieved are appropriate small molecules or polymers *via* dehydration or coupling. These manufacturing procedures are typically uncontrollable that leads to the heterogeneity in the aspect ratio of GQDs. The admirable thing is that the GQDs are biocompatible because of the usage of non-toxic reagents. As per the available knowledge, most of the synthesis process through the top-down approach involves cleavage of carbonaceous material. However, these methods have major drawbacks in terms of low yield, unexpected damage in structure and non-uniform morphologies.^26–28^ Owing to the unique structure and excellent properties of GQDs, the main top-down approaches designated till date are, oxidative cleavage, hydrothermal or solvothermal method, microwave-assisted or ultrasonic-assisted process, electrochemical oxidation, chemical vapour deposition (CVD), pulsed laser ablation (PLA) and electrochemical method.^29–38^ On the other hand, bottom-up approaches offer controllable synthesis and carbonization. The synthesized GQDs have a good range of size, strong luminescence emission and satisfactory properties.^39–55^

### 2.1 Top down methods

#### 2.1.1 Liquid exfoliation

**Fig. 3:**
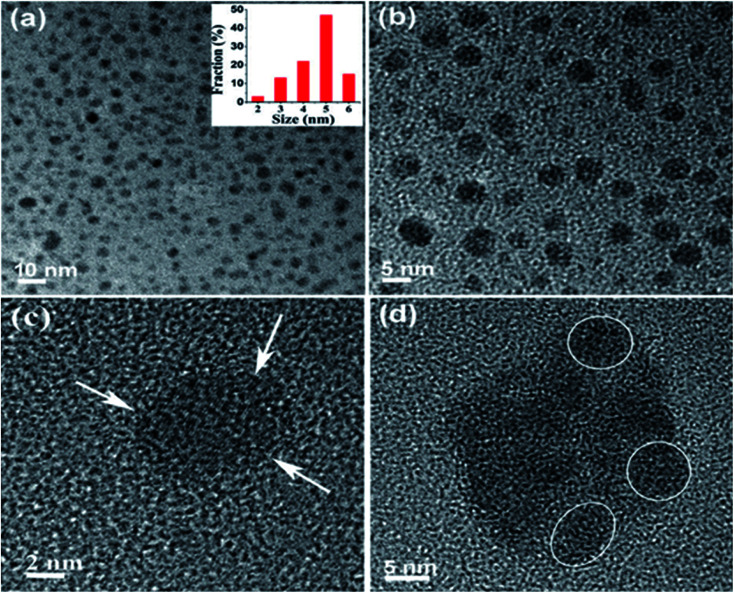
(a and b) TEM images of GQDs in water, inset shows size distribution of GQDs in water. (c) HRTEM image of a single GQD. (d) GQDs in DMF show slight agglomeration. Reproduced with permission from ref. 57, copyright 2016, The Royal Society of Chemistry.

Liquid exfoliation (LE) of the 2D materials has drawn huge interest due to the scalability. LE through ultrasonication is the most capable behaviours to yield nanosheets with numerous benefits like low-cost fabrication, ease in operation and minimize environmental impact. During this process, if the graphite as a precursor is exfoliated to graphene layers then GQDs with good crystallinity can be prepared by the LE method. To prepare GQDs, low and highly defected (edge and surface defects) precursors are graphite and acetylene carbon powder respectively has been used through this process.^56,57^ Graphene was produced through LE of graphite after intercalation by Viculis *et al.*^58^ and has involved great attention recently. Inspired by this, Sarkar *et al.*^57^ have synthesized GQDs through LE *via* probe sonication treatment of graphite powder. The reaction involved the high-energy ultrasonic waves to cut the graphene sheets into ultra-fine particles or GQDs. The as-prepared GQDs extracted in different water based solvents and DMF and characterized the size of the particles as shown in Fig. 3.^57^ The water extracted GQDs are relatively smaller in size than the particles extracted in DMF. The average size and height of the GQDs were 4.6 nm and 1.1–1.9 nm, respectively and it consist of 2–3 graphene layers with 0.353 nm interlayer spacing. Compared to other methods, this technique does not need a carbon source as precursors which is the advantage of this method.^57^ Also, it can control the physical and chemical properties of the GQDs by altering the parameters of the LE technique.

#### 2.1.2 Hydrothermal method

Hydrothermal method is a promising technique to prepare GQDs from the carbon-based starting materials with strong oxidizing agents like HNO_3_, H_2_SO_4_, H_2_O_2_ which are used to breakdown the carbon nanoparticles into GQDs. The advantage of the hydrothermal synthesis of GQDs is that by applying different hydrothermal temperature, GQDs particle size can be varied. In other words, increasing the hydrothermal temperature, the size of the GQDs particle decreased.^59^ For example, Tian *et al.*^60^ have prepared GQDs through the hydrothermal method using GO as a carbon source and H_2_O_2_ as a reagent. According to their mechanism, H_2_O_2_ dissociates into OH radicals while high temperatures and then thermally cut graphite sheets into fragments as the hydrothermal reaction carries on. The experimental results suggest that the graphite disintegrates at increased temperature. Pan *et al.*^61^ developed a hydrothermal route to prepare GQDs that exhibit blue luminescence. Concisely, the graphene sheets were cut into tiny pieces by controlled oxidation in a mixture containing H_2_SO_4_ and HNO_3_ under ultrasonication. The oxidized tiny graphene sheets were then reduced under hydrothermal conditions in a Teflon lined autoclave at elevated temperature. The obtained GQDs had an average diameter of 9.6 nm consisting of 1–3 layers of graphene and exhibited a quantum yield of 6.9% using quinine sulfate as a reference. Shen *et al.*^62^ synthesized functionalized GQDs using polyethylene glycol (GQDs-PEG) *via* a one-pot hydrothermal route. GO sheets and PEG were used as starting materials. The resultant monodisperse GQDs-PEG exhibited a uniform diameter in the range of 5–25 nm. The prepared GQDs-PEG demonstrated excellent luminescence properties when compared to bare GQDs and also the PL quantum yield of the GQDs with 360 nm emission was about 28% using rhodamine B as a reference.

#### 2.1.3 Electrochemical method

**Fig. 4:**
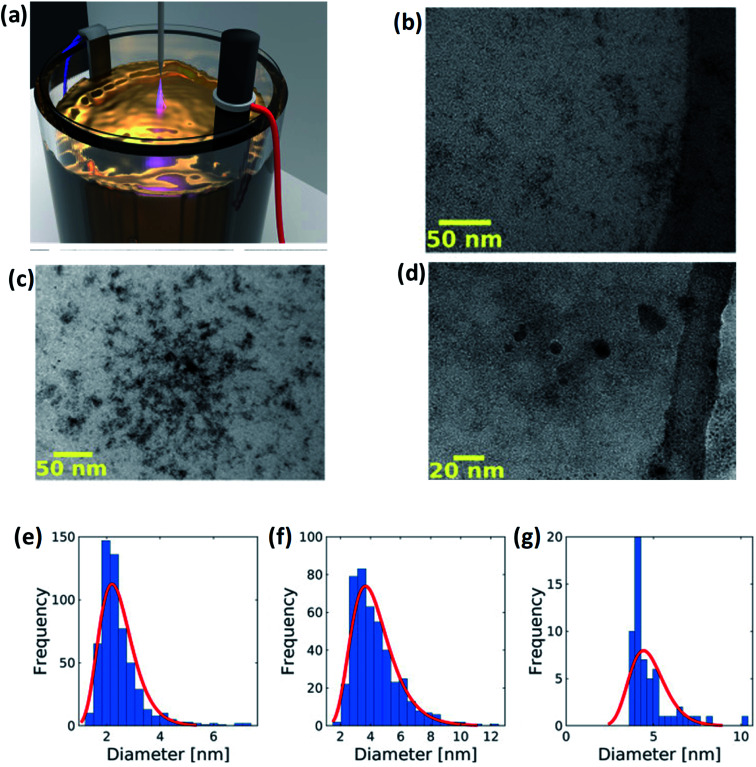
(a) GQDs are fabricated *via* a microplasma-assisted electrochemical process. An electric potential is placed across a graphite rod and titanium electrode submerged in an electrolyte solution of glucose and deionized water. Five kilovolt is placed on a stainless steel probe positioned 1 mm above the surface of the solution. This results in a microplasma discharge that splits glucose and water molecules, freeing up carbon, hydrogen, and oxygen atoms for bonding to the exfoliated GQDs. (b–d) TEM images of GQDs and (e–g) corresponding size distribution of of GQDs at different concentrations. Reproduced with permission from ref. 64 copyright 2019, Elsevier.

Peng *et al.*^63^ have synthesized yellow-green PL emission GQDs through low-cost electrochemical method. The detailed synthesis process of GQDs consists of a NaOH aqueous solution prepared as the electrolyte. Then, a graphite rod and Pt foil acting as an anode and counter electrode, respectively, were immersed in the aqueous solution as shown in Fig. 4.^64^ The process set for 6 h after applying the voltage of about 5.0 V. During the reaction the colour of the homogeneous solution changes from brown to black. The solution was then filtered and finally, the resultant product was GQDs. In another study, Joffrion *et al.*^64^ have been implemented the electrochemical method assisted with microplasma, so-called microplasma-assisted electrochemical method. The authors produced GQDs with glucose precursors *via* electrochemical method with low yield and low intensity of GQDs. To overcome this limitation, the microplasma is added into the electrochemical process so that the electrical current flowing through the system increases. This indicates that the removal of carbon increases by reduction of the glucose molecules. Due to this process, a large amount of GQDs are produced.

### 2.2 Bottom up methods

#### 2.2.1 Microwave-assisted hydrothermal method

**Fig. 5:**
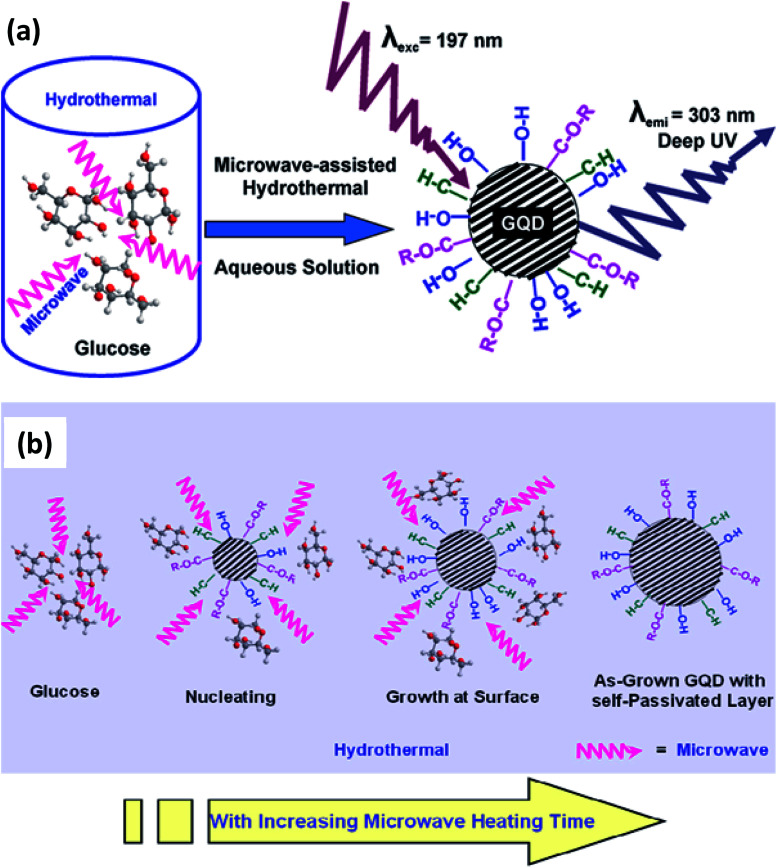
(a) Schematic diagram of synthesis of GQDs *via* microwave-assisted hydrothermal (MAH) technique and (b) schematic diagram of mechanism of formation of GQDs with functional groups. Adapted from the ref. 69. Copyright 2012, ACS Publications.

The hydrothermal method usually consumes a long time to produce GQDs. Therefore, currently a fast method was oppressed by assisting with microwave *i.e.*, microwave-assisted hydrothermal (MAH) method.^65^ By assisting with a microwave, so called MAH is utilized to synthesize the GQDs that share both the benefits of microwave and hydrothermal techniques.^66^ It was developed by Lau's group to prepare water-soluble GQDs using glucose as a precursor. The heating of microwave that provides simultaneous, homogeneous, and fast heating leads to the formation of the uniform size distribution of quantum dots.^67^ The observed emission energy of the GQDs was 4.1 eV which is the largest energy emission at the shortest emission wavelength compared to other QDs.^68,69^ This was first reported by Tang *et al.*^69^ in 2012 for the GQDs which were excited by a 197 nm laser. Fig. 5a shows that the GQDs are prepared through the MAH technique using glucose precursors.^69^Fig. 5b showed the mechanism of the formation of GQDs with various functional groups without any surface passivation agents or inorganic additives.^69^ The glucose molecules dehydrated to form nucleation crystal and chemically active functional groups attached to the surface of GQDs. The glucose molecules are pyrolyzed and then transformed into GQDs. In another study, monodisperse GQDs were synthesized using GO precursor under microwave irradiation with the parameters applied at 200 °C for 5 min. Then the pH was neutralized by sodium carbonate and centrifuged, the resultant supernatant finally separated as GQDs.^70^ GQDS can also be synthesized by inducing the solvent^71^ and the level of doping.^72^

#### 2.2.2 Soft template method

**Fig. 6:**
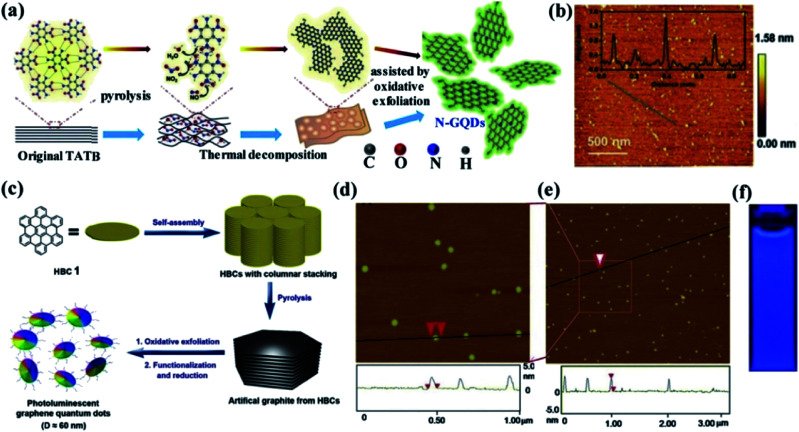
Schematic diagrams and related characterized results of GQDs by using soft-template approach. (a) Illustration of the formation process of GQDs from single-layer 1,3,5-triamino-2,4,6-trinitrobenzene (TATB) intermolecular condensation by soft-template route (b) AFM image of GQDs which was synthesized by using TATB deposited on a freshly cleaved mica surface. Inset: height profile along the dark line in the AFM image. Reproduced with permission from ref. 75, copyright 2016, Wiley online library. (c) Processing diagram for fabrication of GQDs *via* soft-template method based on the carbon source of hexa-peri-hexabenzocoronene (HBC)^76^ (d and e) AFM topography images of GQD prepared by using HBC and height profiles along the line in the images. Adapted from the ref. 76, copyright 2011, ACS Publications. (f) Optical photograph obtained under excitation at 365 nm UV lamp, respectively. Adapted from the ref. 77, copyright 2016, ACS publications.

Generally, the soft template method is an easy and effective method for the production of nanomaterials compared to conventional synthetic methods. This method can successfully control the morphology, particle size and structure of the nanomaterials which is the key advantage. The template method is classified into a soft and hard template according to their different structure.^73,74^ Compared to the hard template method, the soft template method is very much suitable for the fabrication of GQDS. It can afford nanoscale reaction void without any difficulty in separation, purifying processes and also favour the bulk production.^65^ Li *et al.*^75^ have prepared GQDs using a soft template method. In this approach, 1,3,5-triamino-2,4,6-trinitrobenzene (TATB) which is a planar and highly symmetric molecule with six strong intramolecular hydrogen bonds between –NH_2_ and –NO_2_ functional groups exhibiting graphite-like layered structure was used as the carbon source and template. Initially, the TATB was annealed for breaking down the chemical bonds and formation of gases such as NO, NO_2_ and H_2_O during the thermal process. Then, the graphite-like TATB settled into a single layer as a result of the expanding gas. The preparation of GQDs using as-prepared TATB through the soft template method is shown in Fig. 6a while the morphology and size of GQDs is shown in Fig. 6b.^75^ In another study, Mullen *et al.*^76^ have produced monodispersed disk-like GQDs of ∼60 nm by hexa-*peri*-hexabenzocoronene (HBC) as a precursor as well as a template through soft template method as shown in Fig. 6c.^76^ In this process, HBC molecules were stacked like a graphitic framework with defects during the pyrolysis step. The graphite was oxidized and exfoliated with a modified Hummers method and then reduced with hydrazine to obtain GQDs. The thickness and the diameter of as prepared GQDs was 2–3 nm and ∼60 nm respectively as observed from the AFM image as shown in Fig. 6d and e.^76^ The GQDs also exhibited strong blue PL emission under excitation at 365 nm UV lamp as shown in Fig. 6f.^77^ The uniformity and dispersity of the GQDs were characterized by TEM analysis that revealed an average diameter of 2.46 nm and thus the GQDs synthesized through the soft template method exhibited 83% of quantum yield.^78^ Furthermore, it is worth noting that the PL intensity in wavelength range >500 nm was observed to increase with increasing nitrogen content.^79^

#### 2.2.3 Metal catalysed method

**Fig. 7:**
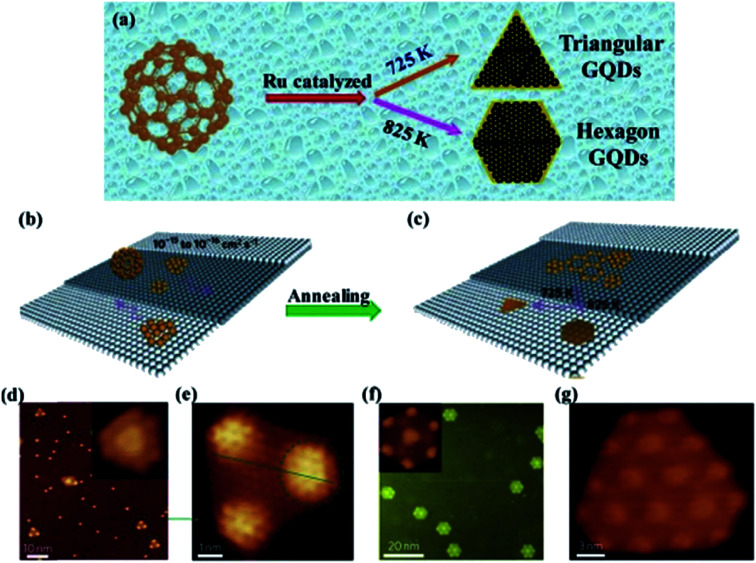
Illustration and STM image of transformation of GQDs through C60 by metal-catalyzed method. (a) Schematic diagram of Ru metal catalyzed cage-opening for C60 under different temperatures. Adapted from the ref. 14, copyright 2015, Springer Publications. (b and c) The majority of C60 molecules adsorbed on the terrace and temperature-dependent growth of GQDs with different equilibrium shapes from the aggregation of the surface diffused carbon clusters; (d) STM image of C60/Ru sample after annealing at 725 K, inset shows magnified view of mushroom-shaped dots. (e) Magnified views of triangular from (d); (fg) hexagon-shaped GQDs obtained after further annealing the sample at 825 K. Adapted from the ref. 81, copyright 2011, Nature Publications.

The metal catalysed method is a rare route for the production of GQDs with low cost and environmentally friendliness.^80^ Lu *et al.*^81^ employed ruthenium as metal catalysed and used C_60_ as a precursor to synthesize GQDs as shown in Fig. 7a. Remarkably, the shape change of the GQDs was observed at different annealing temperature through this metal-catalyzed method as seen in Fig. 7b and c.^81^Fig. 7d–g showed that the triangular and hexagonal shapes of GQDs were observed by annealing the specimens at different temperatures (725 K and 825 K respectively) for 2 min as demonstrated using scanning tunnelling microscopy (STM). Though, the special structure of starting material and a metal catalyst are rarely utilized for the production of GQDs in this method.^81^

### 2.3 Green synthesis method

**Fig. 8:**
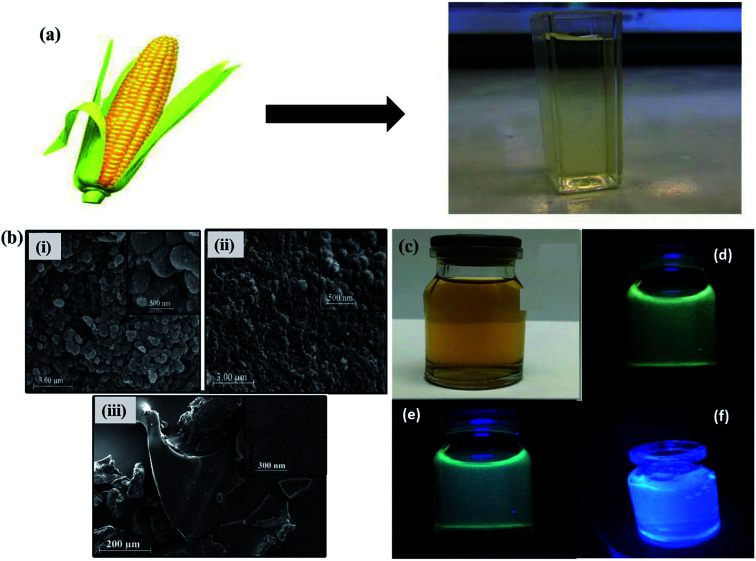
(a) Schematic diagram of GQDs synthesis through green chemistry (b) SEM images of as-prepared GQDs, TiO_2_ and GQDs/TiO_2_ composite, (c) as-synthesized GQDs under normal light and (d–f) UV light. Reproduced with permission from ref. 85, copyright 2017, Elsevier.

Apart from the several synthesis methods as discussed in the previous section, researchers have been seeking to unveil and attain novel production methods for GQDs. The synthesis of GQDs has been highly concerned to obtain the unique characteristics and properties. All the preparation approaches mentioned in the previous section are the fruitful effort to cautiously improve eco-friendly, low toxicity alternatives for semiconductor quantum dots. Although it has high efficiency, the real struggle is in eliminating the by-products such as inorganic salts and acids. Also, it is nearly impossible to apply these approaches in the large-scale production of synthesized material with good crystallinity. As compared to semiconductors QDs and organic dyes, GQDs are eco-friendly, relatively non-toxic and photostable. GQDs have been synthesized through the methods of green synthesis with the help of different carbon precursor sources like fruit extracts, peels, food-wastes, algal blooms, bacteria, milk, cabbage and human urine.^82–86^ When compared to the conventional synthesis, the green chemistry methods have a lot of variance in morphology and UV-vis absorbance. Teymourinia *et al.*^85^ synthesize GQDs through the green chemistry method using corn powder as a precursor as shown in Fig. 8a. The synthesized GQDs were tested against photo-degradation of Rhodamine B (RhB) under UV light irradiation. Compared to conventional TiO_2_ material, GQDs/TiO_2_ shows excellent photocatalytic activity in the degradation of RhB which is about 53% in 80 min. Fig. 8b shows the SEM image of GQDs, TiO_2_ and GQDs/TiO_2_, respectively. It indicates the size of the particles in the range of 20 to 100 nm. Fig. 8c exhibited the emission of different colour under normal light and UV light by controlling the temperature and reaction time.^85^ Furthermore, Chen *et al.*^87^ produced GQDs by green and effective hydrothermal methods using natural polymer starch as a precursor. The resultant products were GQDs, water and carbide precipitate while the diameter of GQDs were in the range 2.25 to 3.50 nm. Anooj *et al.*^88^ prepared GQDs using Nutmeg seed by a green and hydrothermal process by applying temperature of 150–200 °C for 6–10 h and investigated its physicochemical properties. The resultant GQDs showed strong optical absorption in the UV region in the range of 260–320 nm. Table 1 summarizes different precursors, methods and experimental parameters used for the synthesis of GQDs through green chemistry. The major advantage of using such precursors lies in the fact that they are easily available, easy to handle and mostly non-toxic. By using the green chemistry method, carbonization and functionalization can be achieved by employing biomass carbon source and low reaction temperatures with an exhibition of characteristic fluorescence depending upon the functionalities present on the surface.^82–86^

**Table 1:** Summary of GQDs preparation through green chemistry

S. no	Precursors	Methods	Parameters	Ref.
1.	Mango leaves (*Mangifera indica*), ethanol	—	Heated under 900 W, centrifuge at 8000 rpm, dried at 65° for 24 h	82
2.	Coconut husk, millipore water	Hydrothermal	200° for 3 h, centrifuge at 15 000 rpm	83
3.	Salicylic acid, pyridine-2,6-dicarboxylic acid, ultrapure water	UV irradiation	50° for 2 h under UV light the cooled to 5 °C	84
4.	Corn powder, DI water	Hydrothermal	180° for 8 h, centrifuge at 14 000 rpm	85
5.	Cabbage, ultrapure water	Hydrothermal	140° for 5 h	86

## 3. Photoluminescence

Optical properties of GQDs have been thoroughly investigated using PL spectroscopy and absorption in the visible as well as UV regime. Due to the easy captive nature of GQDs and appearance, they reveal distinct absorption and luminescence features. GQDs are very frequently expressed a strong absorption peak in the profound UV region, which is originated from π–π* transition. Also, the small absorption peaks attributing to n–π* transition can be observed at a higher wavelength. The GQDs hold the graphene core and indeterminate chemical groups on their surface and thus the photoluminescence is organized together by graphene core and neighbouring chemical groups.^25^ The basic properties of the GQDs are mainly dependent on the shape, size, edge structure and it play an active role in the positioning of absorption peaks.^90,91^ The other influential factors include functional groups, solvent and temperature which are exposed to induce effects of the basic properties.^92^ The major contributing PL sites on the sample can vary while the wavelength of the excitation light changes.^19^ GQDs emits different coloured photoluminescence contingent to their different synthesis methods, sizes, layered structure, chemical functionalization of their surfaces and better crystallinity as shown in Table 2. Many researchers report that the bandgap of GQDs is induced by their size and hence it can alter their PL. As the bandgap increases as the size of GQDs decreases.

**Table 2:** Summary of morphologies and quantum yields of GQDs obtained through different synthesis routes

S. no	Synthesis route	Diameter of the obtained GQDs	Layers contain	PL QY	Colour of PL	Ref.
1	Solvothermal method	Average 5.3 nm and height 1.2 nm	—	11.4%	Strong green luminescent	19
2	Solvothermal method	3.783 ± 0.829 nm	—	17.4%	Strong green fluorescence	24
3	Photochemical reduction reaction	—	—	8.8%	Blue luminescent	26
4	Ammonia –mediated bond–scission reaction	∼2.5 nm	Single layers (∼1.13 nm thickness)	46%	Different colour emits in a single wavelength	34
5	Hydrothermal	5–13 nm (average 9.6 nm), height between 1 and 2 nm	1–3 layers	6.9%	Blue luminescent	61
6	Hydrazine hydrate reduction	5–19 nm (average 13.3 nm)	—	7.4%	Strong blue & green luminescent under 365 & 980 nm radiation respectively	29
7	Electrochemical oxidation	3–5 nm and height between 1 and 2 nm	—		Green luminescent	89
8	One pot hydrothermal	5–25 nm	—	28%	Blue luminescent	62
9	Microwave assisted	gGQDs – 2–7 nm (avg. 4.5 nm) & height 1.2 nm bGQDs – no perceptible changes	gGQDs contains single or bilayers	gGDQs – 11.7% bGQDs – 22.9%	Blue and green luminescent	93
10	Nanolithography technique with different temperature conditions	1–4 nm	1–3 layers	—	Different colour emits for respective temperature	94
11	Ultrasonification method	3.4 nm	—	3.4%	Bright blue luminescent	95
12	Ozonation pre-oxide hydrothermal route	2–5 nm	—	3.18–9.48% for different wavelength from ∼355 – ∼440 nm respectively	Blue luminescent	96
13	Chemically method	0.7 nm & 0.5 nm height, height between 1 & 3	Single and multilayers	4.04% for single layer & 2.29% for multilayers (2–6)	Green luminescent for single layer & yellow luminescent for multilayer	97
14	Hydrothermal cutting route	1.5–5 nm (avg. 3 nm) & height 1.5–1.9 nm	2–3 layers	7.5%	Strong green luminescent	98
15	Electrochemical method	5–10 nm	Single layer & thickness <0.5 nm	14%	Strong yellow luminescent	99
16	Chemical exfoliation	4 nm	Single layer	—	Blue luminescent	100
17	Two step chemical route	Avg. 5–6 nm	—	Avg. 4.5%	Blue luminescence	101
18	Ultrasonic assisted chemical oxidation method	—	—	15.8%	Yellow luminescence	102
19	Graphite intercalation compound method	3.5 nm	1–3 layers	22.3%	Blue photoluminescence	103
20	Hydrothermal method	4.2 nm	1–3 layers	9.4%	Strong blue luminescence	104
21	Modified hydrothermal route	3.52 ± 0.5 nm	—	30%	Blue luminescence	105
22	One pot hydrothermal method	3.5 ± 0.5 nm	1–5 layers	41.9%	Bright blue luminescent	106
23	Hydrothermal method	5–20 nm	—	53%	Purple luminescence	107

**Fig. 9:**
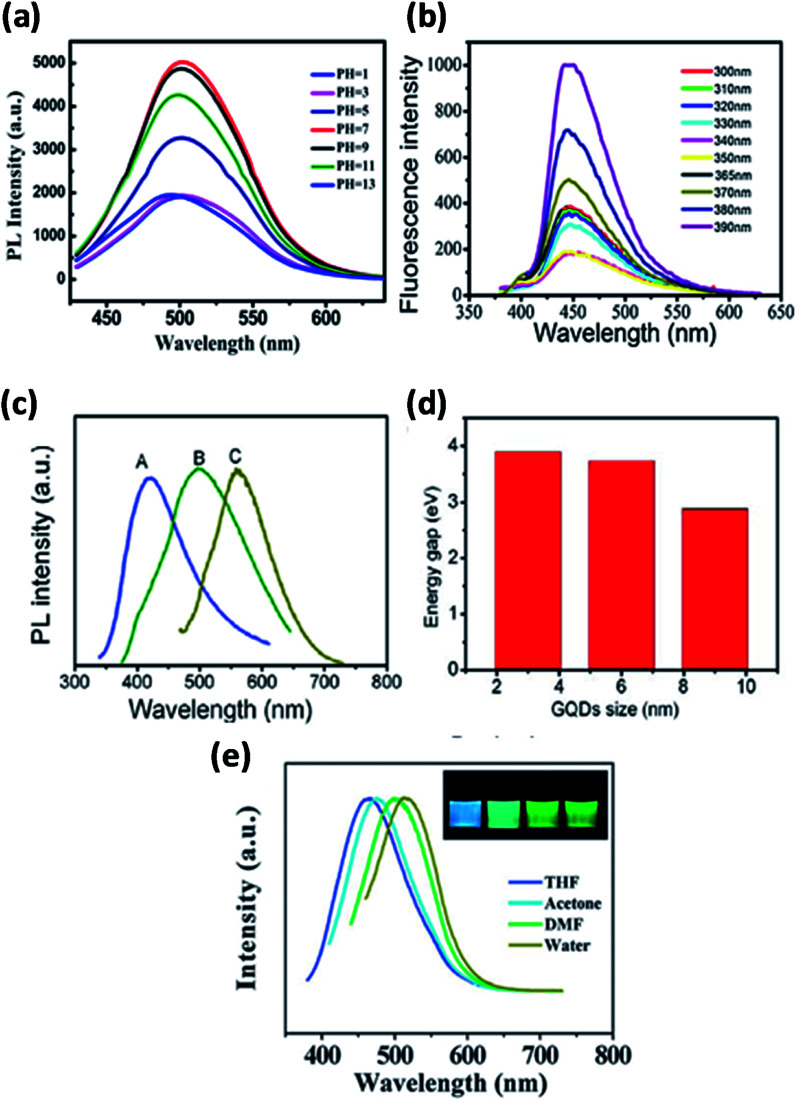
(a) Fluorescence spectra of GQDs at different pH. Adapted from ref. 110, copyright 2015, PLoS One publication. (b) Different excitation wavelengths. Reproduced with permission from ref. 111, copyright 2018, Elsevier. (c and d) Different size of GQDs and their band gap energy. Adapted from the ref. 83, copyright 2016, American Scientific Publisher. (e) Different solvents. Reproduced with permission from ref. 21, copyright 2011, The Royal Society of Chemistry.

The PL emission can vary due to the variations in the size of bandgap and chemical functionalization. GQDs exhibits higher photoluminescence as compared with other carbon-based materials.^8,108,109^ Fundamentally, GQDs have exhibited PL due to the variation in various parameters of the synthesis like concentration, size, pH, solvent and excitation wavelength as shown in Fig. 9 where the dependency of PL has been acknowledged mainly to quantum confinement, edge effect, composition, structure and shape. Fig. 9a and b represent PL spectra at different pH and excitation of GQDs respectively.^110,111^ The colour of the PL spectra given in Fig. 9c is associated with the sizes of GQDs and Fig. 9d represents the energy bandgap with respect to different sizes of GQDs.^94^ The intensity of PL of GQDs is also revealed to be sensitive to the solvents as shown in Fig. 9e. The peak shifted from 475 to 515 nm in THF, acetone, DMF and water, respectively. The insert of Fig. 9e represents the snap of GQDs illuminated by UV light.^21^ Generally, shifting excitation wavelength is dependent on the emission wavelength and the height of the band is characteristically found in photoluminescence spectra. By large the shifting excitation wavelength also shifts towards the higher wavelength of the emission wavelength.^8^

## 4. Quantum yield

Generally, quantum yield is defined as the percentage of absorbed light to the emitted light through fluorescence. GQDs are chemically inert; biocompatible and sustainable. However, certain hindrances prevent their hands-on usage in applications like sensing, bioimaging and energy storage devices due to comparatively stumpy luminescence quantum yield, shifting fluorescence emissions and indistinct luminescence mechanisms.^112^ The enlightening quantum yield is a growing research area. Thus, a huge amount of effort was applied to surge the quantum yield of GQDs over surface chemistry. Surface variation of GQDs cannot significantly expand their quantum yield but correspondingly they can efficiently tune their photoluminescence which primarily contains external oxidation and modification with organic fragments. Usually by changing the electron density of dots they can enact as reaction sites and alter the PL emission due to the functional groups on the surface. In recent years, the achievement of quantum yield has been reported to be between 2 to 46% in GQDs and functionalized GQDs depending upon the individual method of synthesis.^112–114^ Tetsuka *et al.*^34^ succeeded in getting maximum quantum yield (46%) with a single layer of GQDs. Dong *et al.*^97^ achieved a low quantum yield of 2.29% for multilayer GQDs while Kharangarh *et al.*^107^ reported fluorescence quantum yield up to 53% for magnesium and sulphur doped GQDs (Mg–S/GQDs) which is considerably advanced than the reported PL for expectable GQDs. The quantum yield difference is highly dependable on the morphology. GQDs have debris on their surfaces that are enriched with oxygen contained groups which acts as non-radiative electron–hole pair recombination centers. Consequently, the detachability of the oxygen groups may be removed from quantum yield with surface functionalization.^2^ In another study, GQDs samples were tested for quantum yield using fluorescence spectroscopy with quinine sulphate as a reference. The formula used for calculation of the quantum yield is as follows:1
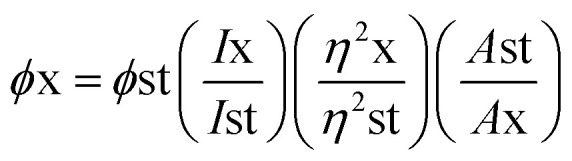
where, *ϕ* is the quantum yield, *I* represent the integrated emission intensity, *η* is the refractive index of solvents, *A* is the UV absorption at 425 nm, *x* is the GQDs, and st is the standard controls.^107^

## 5. Applications of GQDs

### 5.1 Sensors

**Fig. 10:**
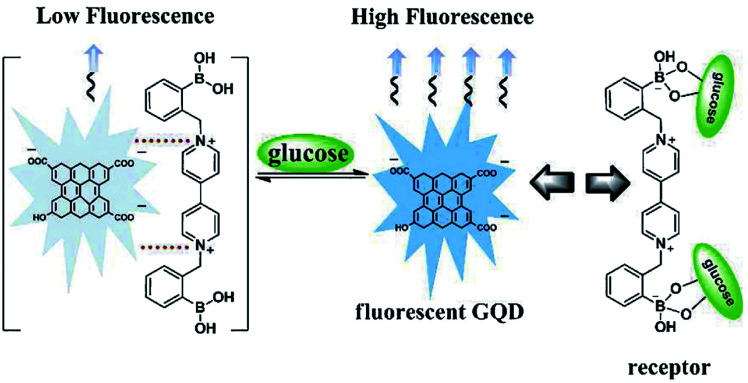
Sensing mechanism based on BBV receptor and fluorescent GQDs. Reproduced with permission from ref. 116, copyright 2013, The Royal Society of Chemistry.

Basically, the sensor is a device that can measure or detect the concentration of molecules in the environs and convert it into an electronic signal to be displayed on the monitor. Investigators have developed a tremendous interest in GQDs owing to their distinctive properties like the fixed bandgap utilized in many applications. It has been recognized that GQDs can act as good sensing material because of their high electron motion with high-speed reaction making them very good contenders for sensing applications. GQDs have been previously examined for field-effect transistors, photovoltaic, light-emitting diodes, electrochemical sensors, glucose sensors, PL sensor, ECL sensor, bioimaging, and bio-labelling.^82,115,116^ A glucose sensor is the general detecting framework working under natural conditions utilizing diverse fluorescent GQDs that contains polar surface gatherings of carboxyl and hydroxyl groups, and a cationic boronic corrosive substituted bipyridinium (BBV) salt. In this sensor, GQDs executed as a fluorescent component, and BBV functioned as a fluorescence quencher and a glucose receptor as depicted in Fig. 10.^116^ The fascination between the GQDs and BBV prompted a reduction in the PL power of the GQDs. At the point when treated with glucose, the boronic acids were changed over to tetrahedral anionic glucoboronate esters, which adequately killed the net charge of the cationic bipyridinium. In this way, the extinguishing productivity was reduced and the PL power of the GQDs was recovered. Thus, taking advantage of the observed PL change, the quick, delicate and specific identification of glucose was figured out.^116^

#### 5.1.1 Photoluminescence sensor

GQDs, possess an excellent combination of properties derived from both graphene and QDs. GQDs have generated large research scopes in the branch of chemistry and biology. Recently, the photosensitive method was used as an encouraging technique to recognize the metal ions by studying fluorescence of raw and surface modified GQDs. While several advantages of GQDs as a sensing component to detect the metal ions have been successfully achieved, the relatively poor quantum yield of GQDs has limited its detection sensitivity. To overcome this drawback an idea was evolved by doping of heteroatom in GQDs such as nitrogen (N), boron (B), sulphur (S), and phosphorus (P) to enhance the quantum yield.^106^ Selectivity is also an important parameter for sensing applications. The selectivity of GQDs and functionalized GQDs was evaluated with various metal ions such as Hg^2+^, Cu^+^, Ag^+^, Fe^3+^, Mn^2+^, Cr^3+^, Mg^2+^, Al^3+^, Co^2+^, Cd^2+^, Ni^2+^.^108^ Many researchers have nurtured the advancement of the development of sensors which is dependent on the sole properties of GQDs. The doping or functionalized GQDs were used for the detection of metal ions, small organic molecules and biomaterials with upgraded selectivity, sensitivity and specificity.^117^ The modification of the optical properties of GQDs such as adsorption of the ions or molecules on the surface can alter the bandgap resulting in PL satiating or enhancement effect. These can be well sensed and in turn, harnessed to create sensors.^118^ GQDs has been broadly studied as a potential candidate in sensing a range of analytes comprising of extraordinary sensitivity and selectivity. Unlike other QDs which are harmful to health, GQDs are non-toxic material. They are highly soluble and possess comparable electronic and optical properties.^66^

The detection of metal ions and nitro compounds that are harmful to the environment and human health have been carried using GQDs as a sensing component doped with heteroatom.^119^ Most of the researchers had used the method of fluorescence intensities quenching effect to detect the presence of metal ions. For example; Hua *et al.*^119^ reported the determination of Hg^2+^, a harmful metal in Campus lake water using GQDs and compared the determination of Hg^2+^ using GQDs coated on the silica surface with CdTe QDs. The fluorescence emission at 465 nm was observed due to the presence of GQDs on the silica surface. GQDs were quenched totally in addition of 50 μM Hg^2+^. As compared to the GQDs, CdTe QDs remain unchanged at 650 nm emission. Liu *et al.*^120^ proposed a successful method for the detection of ascorbic acid (AA) in human blood serum based on fluorescence intensities of GQDs. The optimized results demonstrated good linear and satisfactory response for AA in the detection range and limit of 1.11–300 μM and 0.32 μM respectively. The suggested method was simple, cost-effective and exhibit better sensitivity and selectivity compared with other methods. Caballero-Díaz *et al.*^121^ reported the determination of fenoxycarp in river water samples based on the combination of changing fluorescence intensities in N doped GQDs and AChE as biorecognition elements. The obtained recovery values were in the range of 57.87–77.11% for three different levels of fenoxycarp concentration in samples. Kumar *et al.*^122^ synthesized and investigated nitrogen and sulphur doped GQDs (N, S-GQDs) sensor for the detection of nitroexplosives. Nitro explosives materials are generally 2,4-dinitro toluene (2,4-DNT), 2,6-dinitro toluene (2,6-DNT), 1,3-dinitro benzene (1,3 DNB), 1,4-dinitro benzene (1,4-DNB), 4-nitro toluene (4-NT), 1,2 dinitro benzene (1,2-DNB), 2-nitro toluene (2-NT), nitrobenzene (NB) and many more. The authors achieved 92% of fluorescence quenching effect of N, S-GQDs using 90 μM solution of nitroexplosive. Also, the calculated detection limit was 19.05 ppb. Shiyue *et al.*^123^ reported a metal-ion sensor based on the fluorescence quenching effect of sulphur doped GQDs (S-GQDs) for the detection of silver ions (Ag^+^). The authors attained fast response, detection range (0.1–130.0 μM) and detection limit of 0.03 μM. Xu *et al.*^124^ have successfully synthesized the N-GQDs for the determination of the Fe^3+^ based on the quenching effect of fluorescence intensities of GQDs. Excellent response with a decent linear range between the concentration and fluorescence intensity of the Fe^3+^ was observed within a wide range between 1.0 μM to 2.0 mM and detection limit of 70 nM. In another study, Zor *et al.*^125^ reported novel multifunctional composites based on silica beads, GQDs and molecularly imprinted polypyrrole (PPy) for detecting pesticides. The synthesized novel composites were utilized as a rapid, simple and sensitive platform for the detection of pesticides in a complex medium such as seawater without any prior sample treatment.

Kaur *et al.*^126^ have fabricated N-GQDs using green, facile thermal pyrolysis technique. N-GQDs can be used for the detection of trinitrophenol as nitroexplosive compounds based on the fluorescence quenching of N-GQDs. The authors calculated the detection range and detection limits as 0–4 μM and 420 nM respectively. Tabarak *et al.*^127^ have prepared high luminescence N-GQDs and used for detecting the Ag^+^ which is otherwise toxic to the environment and the human body. The linear range of N-GQDs was observed to be 0.2–40 μM and the limit of detection was 168 nM. Later, this method was used to test the river and tap water with good results. Wang *et al.*^128^ reported an effective fluorescent nanosensor based on GQDs for selective and sensitive determination of Cu^2+^ in the rat brain. The nanosensor shows the detection range from 0.1 μM to 1.0 μM with a limit of detection of 0.067 μM. Anh *et al.*^106^ developed a highly selective and sensitive N, S-GQDs nanosensor for hasty and strong recognition of Hg^2+^ in wastewater by changing the fluorescence intensity of nanosensor. This study reported the detection range and detection limit as 0.05–15 μM and 0.14 nM respectively. Liu *et al.*^115^ developed a novel biosensor to sense the AA and Paracetamol (PAR) based on fluorescence “turn Off–On” of PPy/GQDs composites. The authors optimized a good linear relationship between the relative fluorescence intensity and the concentration of analytes. The obtained values of the AA and PAR are in the range of 3.33–997.5 μg L^−1^ and 0.067–233 μg L^−1^ respectively.

#### 5.1.2 Electrochemiluminescence sensor

Electrochemiluminescence (ECL) is a powerful technique which combines electrochemistry and chemiluminescence and results in electrogenerated chemiluminescence.^129^ Generally, it is based on the emission of light from an excited state formed in the progress of electron transfer processes in the middle of the radical cations and anions of a luminophore. It converts the electrochemical energy into radiative energy through an applied potential on the surface of an electrode.^130^ During the electrochemical reaction, the luminescence signal can be found from the excited state of the ECL luminophore produced by the electrode.^131^ The most striking advantage of ECL is that it does not require the usage of exterior light sources. The position and time of the ECL emission are controlled by variation of the electrode potential. The other important aspects associated with it are high sensitivity along with low cost and small size instrumentation. ECL can be more effective than chemiluminescence since the generation of the state can also be selectively organized by changing the electrode potential. The diversified advantages of it are the absence of simple reaction control mechanism, optical background, refined selectivity and sensitivity and wide range of response. GQDs have been used for the detection of some harmful metals based on their ECL activities as shown in Table 3. Chen *et al.*^132^ developed an ECL sensor made of GQDs/peroxydisulfate (GQD/S_2_O_8_^2−^) system to detect the chromium hexavalent (Cr(vi)) which is toxic in nature. The authors developed GQD/S_2_O_8_^2−^ based ECL sensor with various parameters and succeeded in achieving the linear range of 50 nM to 60 mM and a detection limit of 20 nM. Similar kinds of sensors were developed for the detection of Cr(vi) in spiked river water. Chen *et al.*^133^ also developed a facile and novel ECL sensing method using N-GQDs/chitosan film for nitroaniline (NA) sensing with high selectivity, sensitivity and convenience. The authors found that the signals were improved by NA with HCL and NaNO_2_ successfully for linear detection of NA in the range of 0.01–1 μm mol L^−1^ with the detection limit of 0.005 μmol L^−1^.

**Table 3:** Summary of different sensors of GQDs and functionalized GQDs for detection of harmful metals in specified real samples

S. no.	Type of sensor	GQDs	Target analytes	Linear range	LOD	*R* ^2^	Real Sample	Ref.
1	Fluoresence probe sensor	PPy/GQDs	PAR & AA	0.067–233 & 3.33–997.5 μg L^−1^	0.022 μg L^−1^ & 1.05 μg L^−1^	0.997	Human serum	115
2	Fluoresence probe sensor	GQDs	Hg^2+^	10 nM to 22 μM	33 nM	0.9965	Lake water	119
3	Fluoresence probe sensor	GQDs	AA	1.11–300 μM	0.32 μM	0.9929	Human serum	120
4	Fluoresence probe sensor	Combination of N-GQDs & AChE	Fenoxycarp	6–70 μM	3.15 μM	0.9941	River water	121
5	Fluoresence probe sensor	N,S-GQDs	Trinitrophenol (TNP)	—	19.05 ppb	>0.991	—	122
6	Fluoresence probe sensor	S-GQDs	Ag^+^	0.1–130 μM	0.03 μM	0.9985	—	123
7	Fluoresence probe sensor	N-GQDs	Fe^3+^	1.0 × 10^−5^ to 2.0 × 10^−3^ M	70 nM	0.9953	Lake water	124
8	Fluoresence probe sensor	N/C-GQDs, hydrogel	Trichlorophenol (TCP)	0.1–20.0 μg mL	0.07 μg mL	—	Drinking water & red wine	105
9	Fluoresence probe sensor	N-GQDs	Trinitrophenol (TNP)	0-4 μM	420 nM	0.9934	Water	126
10	Fluoresence probe sensor	N-GQDs	Ag^+^	0.2–40 μM	168 nM	0.994	River & tap water	127
11	ECL sensor	GQD/S_2_O_8_^2−^	Cr(vi)	50 nM to 60 mM	20 nM	—	River water	128
12	ECL sensor	N-GQDs/chitosan	Nitroaniline (NA)	0.01–1 μmol L^−1^	0.005 μmol L^−1^	0.997	Water	129
13	Electrochemical sensor	MIPPy/GQDs	Bisphenol A(BPA)	0.01–50 μM	0.04 μM	0.9979	Tap & sea water	134
15	Electrochemical sensor	GQDs	miRNA-155	—	0.14 fM	—	Human serum	135
14	Electrochemical sensor	GQDs	Cu^+^	—	3 × 10^−10^ M	0.995	—	39

#### 5.1.3 Electrochemical sensor

Dispersal of natural and industrial organic and inorganic pollutants triggers environmental pollution, especially, the air pollution containing unknown chemicals that can affect the water, farming and human health. Thus, at present, this is the main concern pertaining to environmental issues. The growing anxiety about the dissemination and the sway of chemical reagents from the environment is to develop analytical tools such as electrochemical sensors. The electrochemical sensor is a kind of chemical sensor that converts chemical reactions of the analytes on electrode into electrical signals based on the types of electrochemical sensors and provide the information of its environment. Electrochemical methods for sensing and analysis are very sensitive, cheap and easy to handle which can provide data even in remote locations. Nowadays, a lot of electrochemical sensors are available commercially that fulfill the requirements and are environment friendly.^136^ These electrochemical sensors are comprising of molecular recognition and electrochemical signal conversion components and have a wide range of applications in fields of food analysis, clinical diagnostics, biological process monitoring and environmental monitoring because of their intriguing characteristics like small size, simple operation, low cost, rapid sensitivity/real-time and online assessment.

Recently, nanomaterials with layered structure have acquired attention because of their unique properties and are one of the most gifted immobilized molecules. These layered nanomaterials can be combined with other nanomaterial or polymers to prominently improve the sensitivity, activity of immobilized molecules and stability of the resultant sensors.^137,138^ In parallel with the optical, mass and thermal sensors, electrochemical sensors are eye-catching because of their tremendous detectability, simplicity and low cost. Hence, electrochemical have fascinated great interests in analytical chemistry during the past decades and now they have a foremost position among the currently available sensors. There are three types of electrochemical sensors; these are divided into Potentiometric, conductometry and amperometric or voltammetry due to the measurement of the electrical signal.^139^ Tan *et al.*^134^ developed an electrochemical sensor based on the PPy and GQDs composite electrode for the detection of Bisphenol A (BPA) in water by using Differential Pulse Voltammetry (DPV). The sensor exhibit good response with good linear range and detection limits are 0.01–50 μM and 0.04 μM respectively were reported. Wen *et al.*^39^ have reported a novel method integrating O_3_/H_2_O_2_/ultrasound for the synthesis of high-purity GQDs for detecting the trace element of Cu^+^. The detection limit observed was very low 3 × 10^−10^ M. Hu *et al.*^135^ developed a simple and sensitive electrochemical biosensor to sense the miRNA-155 using GQDs. This type of electrochemical biosensor showed great capabilities in terms of selectivity and sensitivity with low detection level 0.14 fM and direct recognition of miRNA-155 in human serum proving great prospective for clinical detection.

#### 5.1.4 Humidity sensor

The general interest in wearable sensors with low power utilization has developed rapidly. In this way, the scientists manufactured and built up the different sensors in different conditions as indicated by the environment. The humidity sensors have discovered supportiveness because of their different use in various fields, for example, day by day life, wellbeing and drug, nature observing, industry, biology, vehicles, meteorology, prescription, sustenance preparing, *etc.* Humidity plays a fundamental role in the condition that stimulates everything in human life. It has numerous downsides likewise particularly in utilization with respect to businesses and innovations. It has achieved such essential position as a result of the vapours containing exceptionally intuitive dipolar particles that either get consolidated or vanished from the surface even with the slight distinction of temperature (this high extremity has happened because of the changes in electron-negativity of the hydrogen and oxygen atoms). Henceforth, it winds up basic to gauge and control the humidity of the earth. Continuous observing of humidity in numerous fields, for example, soil stickiness checking, bundling industry, semiconductor fabricating, nourishment preparing and therapeutic industry, structural building, electronic taking care of, residential apparatuses and cooling frameworks is essential.^140–143^ For manufacturing humidity sensors, the decision of the material selection is much significant and is likewise very difficult. An enormous number of materials like polymers, metal oxides (MOs), carbon-based materials and their composites have been applied. The investigation of these materials that ought to have great sensitivity over the complete range of relative humidity (RH). In addition, the electrical parameters identified with their composites can be shifted because of their screening to humidity.

Recently, humidity sensing properties of graphene-related materials were discovered and fast responses with variation in resistance, capacitance and adsorption of water were perceived. The oxygen-containing molecules at the surface play an excellent role in humidity sensing. An enormous quantity of them may cause the material to act as an electrical insulator, as in the case of GO. The high resistance is not desirable for the sensors based on resistive transduction. GQDs contain not only the characteristics but also the tunable bandgap of graphene.^116,144–146^ By minimizing the size of graphene to several nanometers, the edge effects and quantum confinement can increase the bandgap of the resultant GQDs at the UV regime. The induced size-tunable bandgap together with the high stability and large optical absorption has already made the GQDs as gifted material for applications such as photovoltaic, LEDs and deep UV photodetectors. The conductivity of GQDs might decrease under the photon illumination due to the adsorption of oxygen and water molecules on its surface when exposed to ambient temperature. This phenomenon is called negative photoconductivity (NPC) and can be mainly recognized as the processes of sequential surface adsorbates-induced carrier trapping and photoelectronic de-trapping. Thus, the GQDs are an excellent material exhibiting sensitivity property to the different environmental humidity.^147^ Initially, GQDs were predominantly used in a single electron transistor (SET). Further, sensing control in SETs and GQDs has also been employed to construct electronic sensors for the recognition of pressure and humidity.^10^

**Fig. 11:**
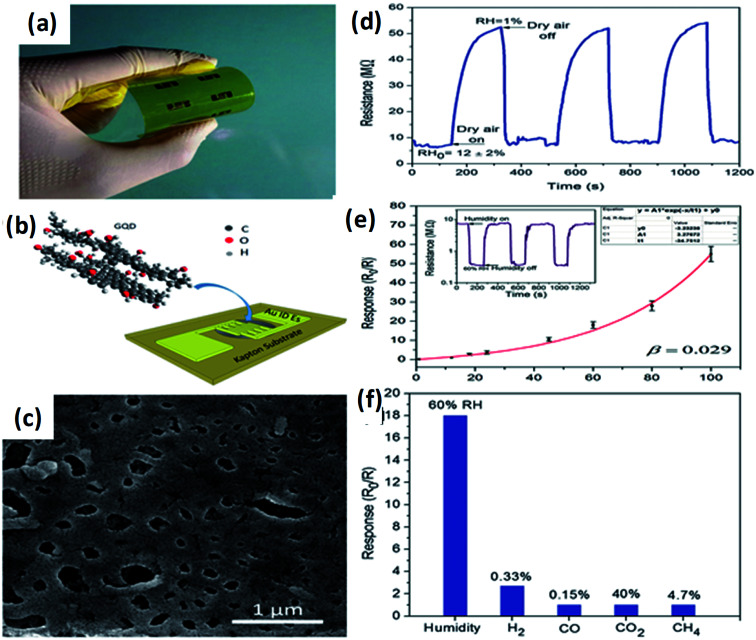
(a and b) Optical image and schematic illustration of the fabricated flexible sensor. (c) SEM image of the drop-casted sensing layer. (d) Transient response of the sensor where the relative humidity controlled by the application of dry air into the sensing chamber. (e) The sensor response to different levels of humidity and inset shows sensor resistance as a function of time upon exposure to 60% RH during subsequent cycles. (f) Response of the GQDs based sensor to humidity in different gases. Reproduced with permission from ref. 143, copyright 2017, The Royal Society of Chemistry.

Recently, Hosseini *et al.*^143^ fabricated highly flexible and sensitive humidity sensors based on GQDs with optimized sensing properties. Good response and selectivity (∼390 for RH change of 99%), broad detection range (1–100% RH), rather short response and recovery times (12 and 43 s, correspondingly), as well as flexibility, was attained. Fig. 11(a and b) shows the schematic design of the flexible humidity sensor on a substrate based on GQDs as a sensing layer prepared by drop-casting. SEM image of the drop casted sensing layer consist of pores and irregular surface as shown in Fig. 11c which may be due to the non-uniform evaporation of water, during drop-casting process of GQDs.^143^Fig. 11d shows the resistance of the sensor corresponding to the time, it indicates the reduction of sensing of the RH by application of dry air at certain time intervals. Fig. 11e shows the sensor response in the range of 1–100% RH and error bars were calculated during repeated cycles. It was observed that the sensor response depends on RH exponentially. The inset of Fig. 11e shows the dynamic response of the sensor to 60% RH during 3 repetitive cycles.^143^Fig. 11f indicates the selectivity of the GQDs based sensor, which results in the sensor responses to CO, H_2_, CH_4_, and CO_2,_ among which the GQDs are highly selective material for humidity sensing of about 60%.^143^

**Fig. 12:**
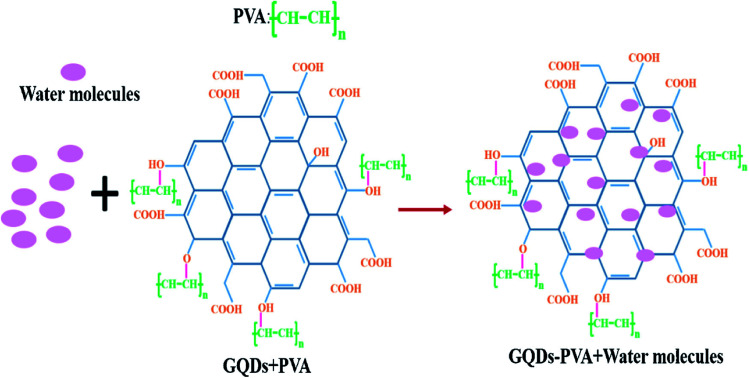
The formation process GQDs–PVA structure with addition of water molecules. Reproduced with permission from ref. 148, copyright 2019, Elsevier.

**Fig. 13:**
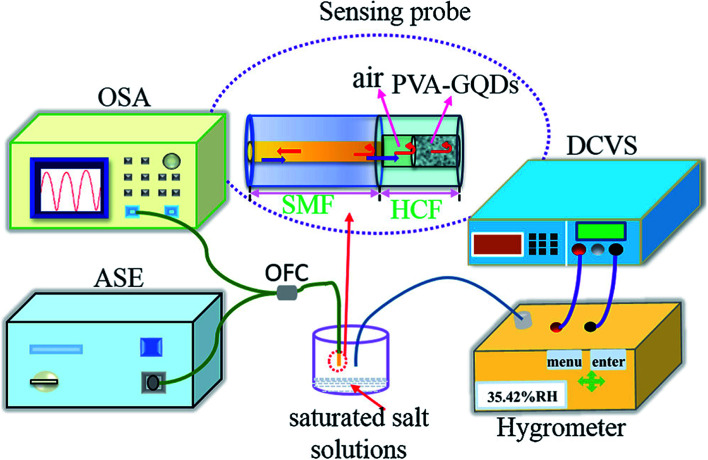
Schematic diagram of the detection system. Reproduced with permission from ref. 148, copyright 2019, Elsevier.

Alizadeh *et al.*^140^ have fabricated a humidity sensor based on GQDs by controlling the carbonization of citric acid and found that the GQDs has an outstanding capability of sensing at the lower RH values to the different atmospheric humidity. RH is the ratio of amount of water vapour present in the air at a given temperature to the amount of water vapour needed for saturation at the same temperature. In another study by Long *et al.*^147^ the GQDs were incorporated with PEDOT : PSS and CNT to form a composite and applied to the humidity sensors. It has been reported that the sensor made from the composite responded well to humidity in the range from 60–80% at room temperature and at atmospheric pressure. The response time and recovery time was about 20 s and 40 s respectively with a small variation in response and recovery time compared to bare GQDs. Yong *et al.*^148^ proposed a most appealing Fabry-Perot interferometer (FPI) in light of GQDs–PVA composite utilized as an RH sensitivity material. The absorption of water changes the estimation of RH. The interaction between PVA and GQDs and GQDs–PVA with water molecules is presented schematically in Fig. 12.^148^ The experiment was executed at 25 °C. Fig. 13 explains the propagation information of input light: broadband (from amplified spontaneous emission (ASE)) propagated from RH sensitive probe where the resultant output light was analyzed by optical spectrum analyzer (OSA) with a spectral resolution of 0.02 nm.^148^ A distinctive RH condition was produced by filling plastic jugs with various soaked saline arrangements, such as lithium chloride, magnesium chloride, magnesium nitrate, sodium bromide, sodium chloride and potassium chloride immersed separately. To produce distinctive RH esteems, a hygrometer was used to record the RH esteemed in plastic jugs, and the hygrometer was connected by the ROTRONIC brand. So as to ensure the exactness of estimation, the RH sensitive sensor test and the test of hygrometer were put at a similar position in the plastic containers. The direct-current voltage source (DCVS) gives vitality to the hygrometer.

**Fig. 14:**
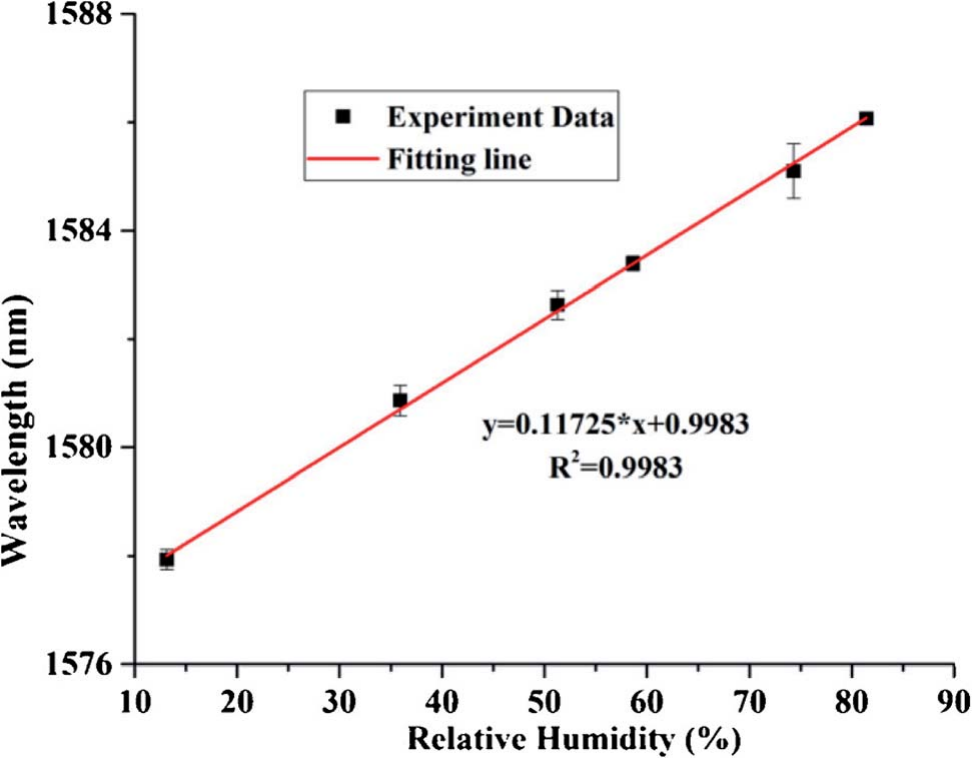
Fitting line between the resonance spectrum and RH values. Reproduced with permission from ref. 148, copyright 2019, Elsevier.

The linear fitting curve of wavelength v/s % RH have appeared in Fig. 14. The uncovered sensitivity was 117.25 pm/% RH in the range of 13.47 to 81.34% RH, in the interim, it keeps up great linearity importance of 0.9983 and resolution is 0.171% RH. The comparison of the current RH sensors based on graphite materials demonstrates that the FPI based on GQDs–PVA combines the benefits of high sensitivity and wide measurements range together. Likewise, RH optical fibre sensor based on GQDs–PVA has a higher connection coefficient than those based on graphite materials. In the interim, the contrasting and fibre stickiness sensors based on different standards shows that the FPI based on GQDs–PVA has numerous points of interest, for example, simple making and high sensitivity. In addition, the great reversibility and repeatability give it more research potential in practical application.^148^ The intrinsic humidity sensing properties based on GQDs in various fields of application are discovered and successfully utilized the uniqueness of the material. GQDs are shown to be immensely sensitive to existing atmospheric moisture and show the variation in resistance with rising RH levels. Such sensor devices would also work at very low RH levels with instant response time and exhibit excellent potential for the development of ultra-small low power humidity sensors.

#### 5.1.5 Gas sensor

**Fig. 15:**
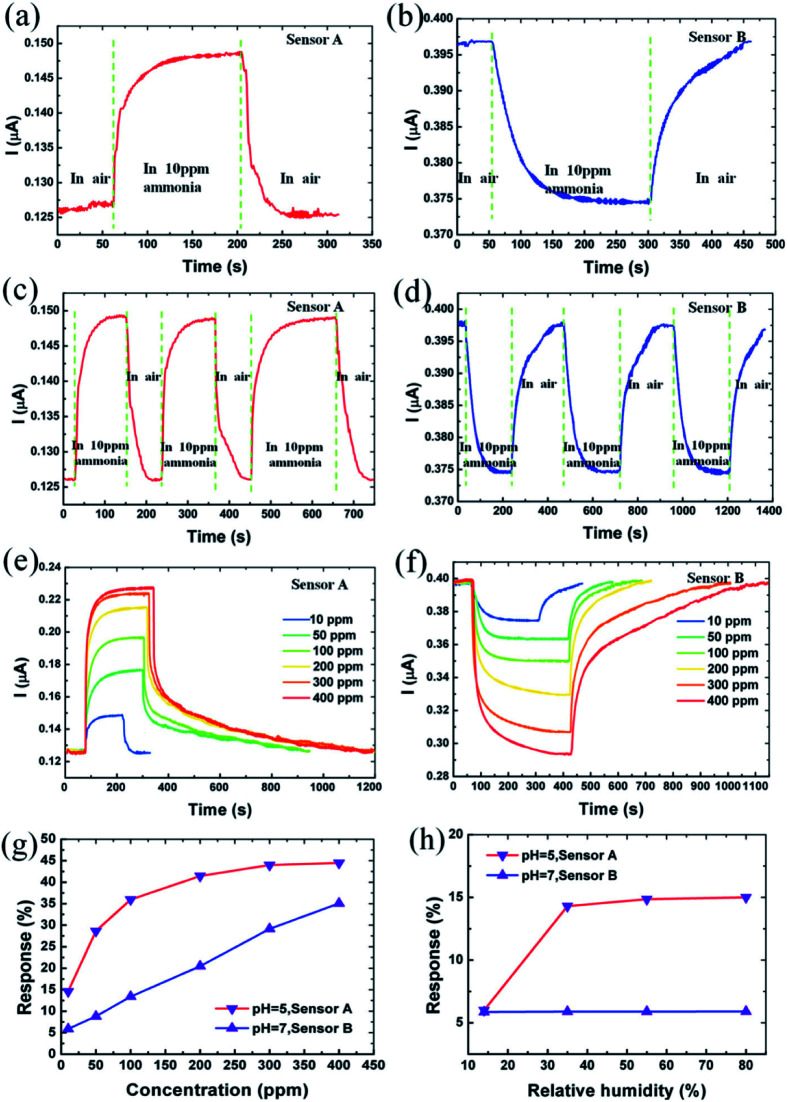
(a and b) 10 ppm ammonia response characteristic of sensor A and B, respectively. (c and d) Response of sensor A and B to 10 ppm NH_3_ for three cycles, respectively. (e and f) Current response behaviour for sensor A and B at different concentrations of NH_3_ ambient (g) absolute response of sensors A and B at various concentrations of NH_3_ (h) absolute response of sensors A and B to 10 ppm NH_3_ at various relative humidity. Reproduced with permission from ref. 150, copyright 2016, Elsevier.

Sensing is one of the environmental remediations particularly employed for monitoring and capturing the evolution of gases from a growing society. During these years with growing greenhouse gas emissions and global warming problems are arising due to the existence of the volatile organic compounds (VOC's). GQDs has been evolved as a sensor material due to more edge atoms than 2D materials because there are more surface atoms therefore more adsorption occurs.^149^ Chen *et al.*^150^ synthesized two different GQDs (neutral and acidic) and utilized for fabricating NH_3_ gas sensors named as sensor A and B. The characteristic studies of sensor A and B were observed with the exposure of different concentrations of NH_3_ gas as shown in Fig. 15.^150^ The current responses are −14.9% and 5.9% when the sensors were exposed to NH_3_ gas as shown in Fig. 15a and b, respectively. It was indicated that the sensors have different electrical responses with the same concentration of the gas molecules. Fig. 15c and d indicates that the responses of the sensors A and B to 10 ppm of NH_3_ for three cycles resulted in the resistance that can recover its initial state after GQDs sensor material allowed into the air from NH_3_. This shows the high stability of the GQDs based gas sensor. Fig. 15e and f shows the responses of the sensor A and B to different concentration of NH_3_ demonstrating that the response and recovery time vary with increasing NH_3_ concentration. Fig. 15g shows the absolute response varying with different NH_3_ concentrations.^150^ The value of absolute response increases with increased concentration of NH_3_ and saturate at higher than 300 ppm. The response was recorded as 44.5% in sensor A, while the response of sensor B varies linearly and recorded as 35.1%. Also, the response of the sensors A and B made with aqueous GQDs with pH 7 and pH 5, respectively is shown in Fig. 15h. Sensor A made with pH 7 shows a slight change in response to 10 ppm NH_3_ under relative humidity from 14% to 80%, while the sensor B made with pH 5 shows changes in response to 10 ppm at low relative humidity.^150^

### 5.2 Biomedical application

#### 5.2.1 Bio-imaging

**Fig. 16:**
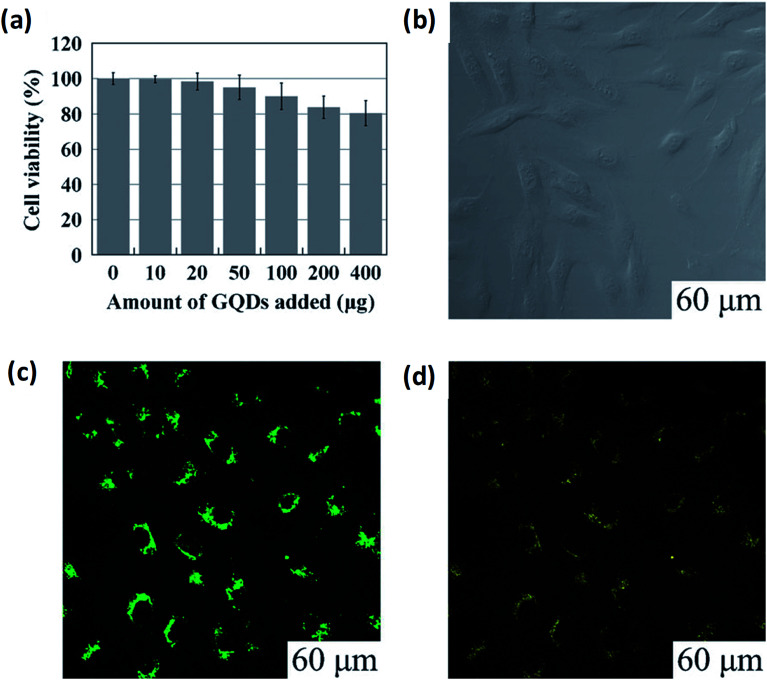
Microscopic analysis for imaging and cellular toxicity of GQDs. (a) Cells viability of MG-63 cell with corresponding amount of GQDs. (b–d) are the images of washed cells under bright field, 405 nm, 488 nm excitations, respectively. Reproduced with permission fromref. 21, copyright 2011, The Royal Society of Chemistry.

Bio-imaging has an important role in both research and clinical application and permits observation and learning of biological processes from subcellular to the small animal level. Investigators were able to find out the initial stage of ailment and to screen the behaviour response with the suitable bioimaging probes.^151^ Recently, GQDs were labelled as a class of fluorescent nanomaterial having unique optical properties that can lead to excellent results based on bioimaging for the diagnosis and treatment of diseases in biological systems (Table 4) owing to their 0D structure, low toxicity, high solubility, biocompatibility and chemical inertia. In the physiological circumstance, GQDs are the most sought after materials these days. For example, Zhu *et al.*^21^ performed experiments by adding up to 400 micrograms along with 150 mL of culture medium (10^4^ cells) which could not weaken the cell activity as suggested by the MTT assay as shown in Fig. 16a. GQDs through the cell's membrane (excitation at 405 nm) were observed using a confocal fluorescence microscope by surveillance of the bright green area inside the cells as shown in Fig. 16b and c. Thus, the bioimaging is dependent upon the excitation behaviour of the GQDs which leads to several visible results dependent on PL. The resultant report was consist of excitation changes in the range of light from green to yellow colour at 488 nm as shown in Fig. 16d.

**Table 4:** Summary of functionalized GQDs synthesis routes, morphology, fluorescent emission and bio-imaging in various biological parts

Functionalized GQDs (F-GQDs)	Preparation method	Emission colour	Bio-imaging on biological cells	Size	Thickness	Ref.
rGOQDs	One step microwave assisted greener route	Green emission at 360–520 nm	Artemiasallina	2–8 nm	—	152
A-GQDs	Two step microwave assisted method	Blue and green at 405 & 750 nm resp.	Human lung carcinoma A549 cell	3–5 nm	—	153
N-GQDs	Ultrasonic shearing reaction method		Bacteria	8.0 ± 0.4 nm	∼0.36 nm	154
RF-GQDs	Facile electrochemical exfoliation of graphite	Red emission at 488 nm	Hela cells in cytoplasm	3 nm	<1 nm	155
E-GQDs	Facile method	Blue emission at 365 nm	RAW 264.7 cells	8.2 ± 1.2 nm	1.5 ± 1.0 nm	156
F-GQDs	—	Green emission	Hepatic cancer cells (HuH-7 cells)	Avg. 7 nm	—	157

In the work of Su *et al.*,^158^ the structure of a peptide with trifunctional themes is accounted as the antecedent obstruct for developing a novel multifunctional protein nanofiber (PNF) and further conjugated with profoundly fluorescent GQDs by noncovalent collaborations. The GQDs basically keep up their good optical properties in the PNF-GQDs nanohybrids. Decent biocompatibility of the PNF-GQDs nanohybrids was found using cell viability tests. With both a recognition moiety and an imaging test, these PNF-GQDs nanohybrids showed the capacity of focusing on and imaging tumor cells at the same time. This examination shows that GQDs–PNF nanohybrids have extraordinary potential as a multifunctional material for biomedical applications, especially, where the capacity of the sensitive tracking and effective labelling is valued. Ge *et al.*^159^ exhibited another photodynamic treatment (PDT) operator dependent on GQDs that can create singlet oxygen (^1^O_2_) through a multistate refinement process, bringing about a quantum yield of 1.3 with the most elevated description for PDT specialists. The GQDs likewise show a wide retention band crossing the UV region and the whole visible region with a strong deep-red discharge. Through *in vitro* and *in vivo* examinations, it was demonstrated that GQDs can be utilized as PDT operators, all the while permitting imaging and giving an exceedingly proficient malignant growth treatment. The present study also prompts another age of carbon nanomaterial-based PDT specialists with performance better than regular operators as far as ^1^O_2_ quantum yield, water dispersibility, photo- and pH-strength, and biocompatibility is concerned.

#### 5.2.2 Biosensing

The optical properties of the GQDs can be utilized for biosensing. In addition to bioimaging, biosensing is also based on the detection of an emitted photon through PL of GQDs. The GQDs assisted biosensor exploits the affinity between analyte biomolecule and GQDs functional group to detect the presence of biomolecule. The ions essential for biological processes or responsible for acute toxicity have to be efficiently transported and regulated at the cellular level. Thus, the sensitivity and selectivity of the *in vitro* ion biosensor is very important. On a similar front, an ethylenediamine modified GQDs (E-GQDs) based Ni^2+^ sensor displayed significantly quenched strong yellow PL emission in the presence of Ni^2+^. The Ni^2+^ detection limit was 3 × 10^−8^ m with a quantum yield of 83% and it's *in vitro* sensing ability was demonstrated by treating adipocyte-derived stem cells in rats.^160^ Increased level of hydrogen sulphide (H_2_S) leads to illness related to cancer and Alzheimer's. A (2,4-dinitrophenoxy) tyrosine (DNPTYR) functionalized GQDs based sensor was reported for H_2_S sensing with a detection limit as low as 2 × 10^−9^ m.^161^ In another study, nitrogen-doped GQDs (NGQDs) were functionalized with tris(hydroxymethyl)aminomethane for the detection of 2,4,6-trinitrophenol (TNP). The senor displayed considerable photoquenching in the presence of TNP due to the overlapping of an emission spectrum of the NGQDs and absorbance spectrum of TNP.^162^ Colorimetric based hydrogen peroxide and glucose biosensor were also reported using nitrogen-doped GQDs as a catalyst.^163^ Apart from *in vitro* studies, the water solubility and biocompatibility of the GQDs make them a suitable candidate for *in vivo* studies. Despite this very few studies explored the *in vivo* sensing capabilities of GQDs. In one such study, *in vivo* detection of the noble metal ions Pd^2+^, Au^3+^, and Pt^2+^ in zebrafish were accomplished using carbon dots.^164^ The authors labelled it as CDs, the optical characterization and physicochemical showed results similar to many GQDs systems. A detailed review of the GQDs based biosensing has been reported by Zhang *et al.*^165^

#### 5.2.3 Drug delivery systems

The outstanding chemical and tunable physical properties, along with ease in distinct surface functionalization make GQDs promising material for drug delivery systems.^166^ The graphene nanosheets size-dependent coupling with DNA molecules was investigated by Zhou *et al.*^167^ The study revealed that the DNA molecule intercalating ability is more visible for small lateral size GQDs.^167^ Doxorubicin (DOX) is a widely used agent in the treatment of human cancers, like soft tissue sarcomas, aggressive non-Hodgkins lymphoma and breast cancer.^168^ For the accurate release of DOX into cancer cells, drug targeting ligand is bound to the functional groups of GQDs and drug is loaded on the surface by π–π interaction.^169^ A DOX/GQDs conjugate system displayed an efficient drug delivery in the treatment of breast cancer cell lines (MCF-7).^170^ A targeted DOX delivery to cancer cells using folic acid as the ligand on GQDs was reported by Wang *et al.*^169^ The inherent fluorescence of GQDs facilitates real-time monitoring, targeted drug delivery and selective cell labelling. Using arginine–glycine–aspartic as a ligand, Qiu *et al.* reported a pH-sensitive, traceable and fluorescent GQDs-based DOX drug release system.^171^ Recently, GQDs-based cell traceable and biocompatible targeted delivery of DOX was reported using biotin as the ligand molecule.^172^ On a similar front, specific drug delivery by GQDs anchored with hyaluronic acid (HA) as the targeting agent was demonstrated by Nahain *et al.*^173^ To examine GQDs assisted DOX targeted drug delivery, innovative real-time monitoring through Forster Resonant Energy Transfer (FRET) was established by Chen *et al.*^174^ In another study, GQDs-based monitoring for targeted delivery of multifunctional core–shell structure loaded with paclitaxel (PTX) anticancer drug was reported by Jing *et al.*^175^ A detailed review of the GQDs assisted targeted drug delivery for cancer treatment was done by Daniela *et al.*^166^

### 5.3 Energy storage

#### 5.3.1 Supercapacitor

The tremendous energy consumption and fast development of technology are the two major factors that mandate for high-performance advanced energy storage devices and technologies.^176^ Subsequently, the conversion and storage of electrochemical energy systems serve as the attractive option and accordingly it has been an emerging research topic among industrial and academic sectors. The electrochemical energy storage systems (EESS) have been used to store the energy converted from chemical energy to electrical energy.^177^ EESS which have engrossed huge interests owing to the high charge–discharge rates and long life-time expectancy are an essential requirement for any energy storage devices.^178^ The supercapacitor is the fundamental device of the EESS that revolves around the great attention and major power sources for the current situation. It is also termed as an electrochemical capacitor and can provide a fast charge/discharge process and delivers long-term cycling stability and high power density. These features enable them to be one of the superior performances giving materials for EESS having applications in the area of portable electronic, electrical vehicles and power back-up systems.^179^ The electrochemical performance of supercapacitors together with capacitance and cycling steadiness are hooked on the composition and structure of their probe materials. Until now, a limited transition metal oxide, conjugated polymers and carbon resources have been established as the energetic storage media.^180,181^ Most recently, numerous studies on the EESS properties of GDQs and their potential applications as electrode materials have been carried out. The GQDs would be encouraging antecedents to formulate advanced energy storage materials because (i) the conjugated carbon skeleton with 0D structure is much stretchy to build complex and conductive architectures, (ii) the enhanced edge structure and functional groups may deliver big amounts of active locations for energy storage and (iii) good chemical reactivity and migration property permits their easy assemblage or process.^177,182^

**Fig. 17:**
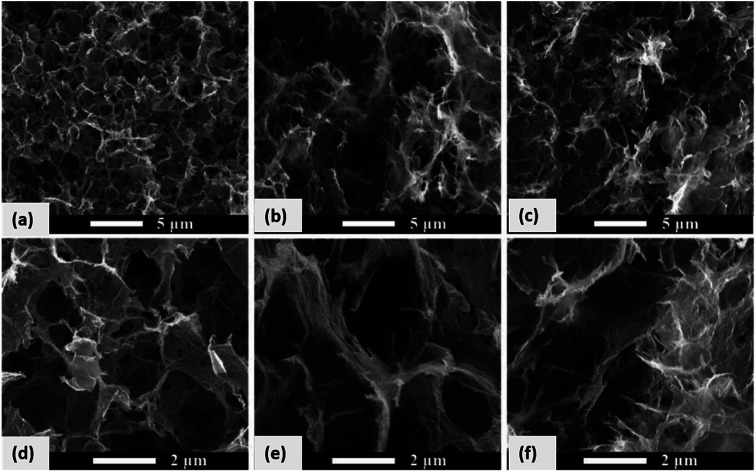
(a–f) SEM images of GQDs/3DG with different composition. Adapted from ref. 183, copyright 2019, MDPI publications.

**Fig. 18:**
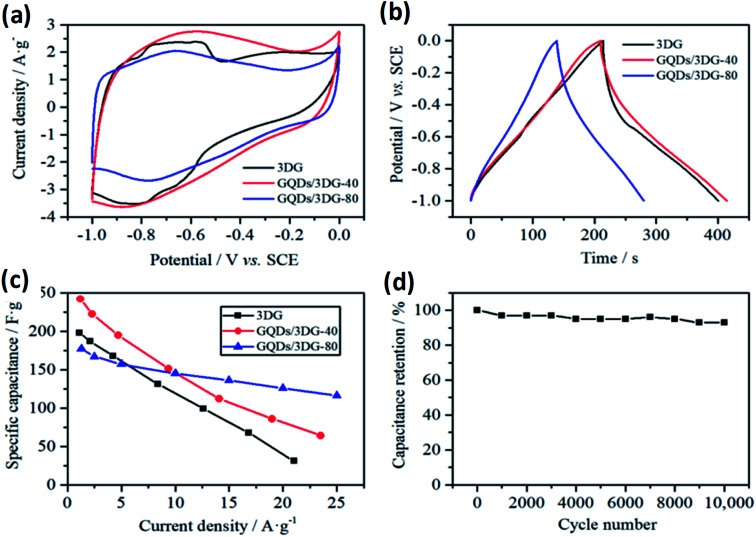
(a) Cyclic voltammetry curve at a scan rate of 10 mV s^−1^ (b) charge/discharge curves at different current densities with different compositions, respectively (c) specific capacitance *versus* current densities and (d) cycling stability at a current density of 24 A g^−1^ for the composite material. Adapted from ref. 183, copyright 2019, MDPI publications.

Recently, some investigators have established that GQDs comprises the required EESS properties which can significantly increase the performance of supercapacitor. The researchers also found that the performance of electrochemical supercapacitor can be enhanced using GQDs composite as compared with unadorned GQDs.^181^ Luo *et al.*^183^ incorporated GQDs into the 3D graphene (3DG) *via* the one-step hydrothermal process and investigated the effect of GQDs in supercapacitors. Fig. 17a–f shows the SEM images of bare 3DG and GQDs/3DG composite. Fig. 17(a and d) indicates the pores present in the pure 3DG with the size distributed from submicrometer to several micrometers. Fig. 17(b and e) indicates that the GQDs/3DG-40 composite material exhibit larger pores and Fig. 17(c and f) (GQDs/3DG-80) indicates the more compact structure than pure 3DG due to the reduced the level of GQDs. Compared to GQDs/3DG-80, GQDs/3DG-40 had a larger surface area. Fig. 18 shows the comparison of the electrochemical properties of pure 3DG and GQDs/3DG as shown in Fig. 18a–d. From Fig. 18a cyclic voltammetry curves indicate that the redox peaks were obscure and nearly rectangular CV curves were presented at the different scanning rates. Fig. 18b shows the charge/discharge curve at the current density of *ca.* 1 A g^−1^. It indicates the composite material had optimal specific capacitance and better electrical conductivity due to the specific surface area of GQDs than pure 3DG. Fig. 18c shows the graph between specific capacitance and current densities of 3DG and GQDs/3DG composite. It indicates that specific capacitance decreases as the current density increases. Fig. 18d indicates cyclic stability exhibiting 93% capacitance retention after 10 000 circles owing to the presence of high-quality GQDs.

Apart from metals, polymers also play an energetic role in supercapacitors especially conductive polymers (CPs) because of their unique advantages such as easy synthesis, flexibility, high electron attraction and redox behaviour. The unique advantages of both GQDs and CPs can enhance the activity of supercapacitors. Recently, Jin *et al.*^184^ fabricated a stable conductive polyaniline (PANi) doped with GQDs and their electrochemical properties. It was found that the changes in the electrochemical properties of GQDs@PANi are strongly dependent on the loading quantity of GQDs in PANi. The maximum specific capacitance of 3632.0 F g^−1^ was reported for applications as an electrode for supercapacitors. Syed *et al.*^185^ has prepared PVA–GQDs/PEDOT nanocomposite and used as a supercapacitor electrode. PVA–GQDs/PEDOT nanocomposite electrode shows a higher current–potential reaction related to bare PEDOT and PVA/PEDOT due to the large surface area of GQDs. This leads to an improvement in the charge accumulation and charge storage. From the results of CV curves of PVA–GQDs/PEDOT nanocomposites at several scan rates, it can be deduced that with the increasing scan rates there is rapid diffusion of electrolyte ion into the surface of the active electrode. Specific capacitance (*C*_sp_) decreases with increasing scan rates owing to the inadequate time of ion to be transferred to the active electrode at a high scan rate. The *C*_sp_ value of 81% was maintained for PVA–GQDs/PEDOT nanocomposites which exhibit good rate capability. High specific capacitance and specific energy of 291.86 F g^−1^ and 16.95 W h kg^−1^ respectively at a current density of 2.0 A g^−1^ was achieved. In addition, the authors study the stability of PVA–GQDs/PEDOT which was found to be much greater than that of PEDOT.

#### 5.3.2 Lithium-ion batteries

The growing concerns of the depletion of fossil fuel resources and environmental issues have admonished people to the needs of the power, which is highly important to improve sustainable energy technologies, renewable energy sources such as tidal, wind and solar energy in the human society. Because of this, the attention of numerous researchers has attracted towards developing efficient energy storage (EES) devices. The reliable ESS such as batteries and supercapacitors are important elements to permit the development of these energy structures.^186^ In addition to conventional lead-acid, Ni–Cd, Ni-MH, and supercapacitors, lithium-ion (Li-ion) batteries are unquestionably a predominant substitute for other energy storage devices. They are capable of diminishing the existing requirement and advancing the application of major energy sources due to their lightweight, high energy and good performance characteristics. In recent years, various progressive Li-ion batteries such as sodium/aluminium, lithium–air/–sulphur and aqueous metal-ion batteries have been emerging, and great efforts have been dedicated to enhancing their overall performance for future practical applications.^186,187^

Generally, a Li-ion battery contains a cathode, anode, electrolyte, an outer case and sealing parts. As of now, many kinds of Li containing cathode materials such as lithium manganese, lithium cobalt oxide, FeS_2_, V_2_O_5_, lithium-ion phosphate, lithium nickel cobalt, manganese oxide, and conducting polymers *etc.* have been investigated. The resources that are characteristically used for constructing the anode are graphitic carbon, metallic lithium, synthetic graphite, hard carbon, tin-based alloys, lithium titanate, and silicon-based materials which are commonly available in the commercial market.^187^ The electrolyte solutions are used such as LiClO_4_, LiPF_6_, LiCF_3_SO_3_ and LiAsF_6_. Apart from these main constituents, there are other components such as flame retardant, a binder, gel precursor and electrolyte solvent. Li-ion batteries have been widely used to control a variety of transportable electronic devices because of their eco-friendly nature and higher energy density. Even though, some of the hazardous heavy metals and organic electrolytes are still being used in Li-ion batteries such as cobalt and flammable organic solvents respectively which can cause severe environmental pollution.^186–188^

Rechargeable Li-ion batteries are very well known these days and can play a much important role in power devices which are portable electronic systems used in the current situation. However, many researchers have faced the problem to achieve the high specific capacity and energy density which is insufficient to meet the increased energy-demanding applications including electric vehicles and grid-level energy storage. To solve these challenges, many researchers try to find an advanced battery system with superior performance over current technologies.^187,188^ Recently, graphene-based materials have attracted much attention due to their exclusive properties that greatly reduce other alternatives for electrode materials for EESS, *i.e.*, lithium batteries, supercapacitors *etc.*^189^ The quantum confinement in GQDs induces the finite bandgap in the material which intern changes the electron conductivity. It is reported that quantum confinement can affect the lithium diffusivity thereby influencing the electrochemical performance and long-term electrochemical cycling of battery.^190^ Fascinatingly, GQDs are extended with oxygen functional groups on their surface, whereby unique properties such as luminescence on excitation and non-zero bandgap have also been well-known. Also, it is expected that GQDs can consistently protect the target material due to their small size. Indeed, GQDs have been specified to serve as a composite or coating material for energy storage devices.^191^

The introduction of GQDs accelerates the large electron transfer and the electrolyte transport of the electrode, leading to enhanced electrochemical performance for Li-ion batteries.^192^ The main advantage while using the GQDs as an electrode coating material is the productions of large surface area for ion transfer between the electrolyte and the active material, thus achieving the ultra-fast energy storage and release.^193^ With the above qualities of GQDs coated on composite with other metal as an electrode exhibits superior high-rate capability and cycling stability in Li-ion batteries. For example, Chao *et al.*^192^ investigated GQDs coated VO_2_ material as an electrode in Li-ion battery with enhanced electrochemical performance. The GQDs layer may work as a surface sensitizer and protector. Thus, the electrode delivers a capacity of more than 420 mA h g^−1^ and capacity retention of 94% after 1500 cycles at 18 A g^−1^. Similarly, Guo *et al.*^194^ studied GQDs/MoS_2_ system as an electrode material which delivered the high capacity (1099 mA h g^−1^ at 100 mA g^−1^), improved cyclic performance and excellent rate capability (660 mA h g^−1^ at 5000 mA g^−1^). Lijuan *et al.*^195^ carried out a facile synthesis of phenylalanine functionalized GQDs (PF-GQDs) and surface treatment of Si nanoparticles. The obtained PF-GQDs@SiNPs was used as an electrode in the Li-ion batteries exhibiting excellent electrochemical performance. The specific capacity was 4066 mA h g^−1^ at 50 mA g^−1^, 3796 mA h g^−1^ at 100 mA g^−1^ and 1820 mA h g^−1^ at 1000 mA g^−1^. In comparison with MoS_2_ and VO_2_, the specific capacity of SiNPs with GQDs as an electrode material offers a more effective range in Li-ion batteries.

### 5.4 Energy conversion

#### 5.4.1 Solar cells

For decades, the rise of novel devices with technologies has provoked accumulation and effectual consumption of energy. This has enacted as cheering to discover innovative ways for the production of clean energy. Solar has a rich, inexpensive, harmless and uncontaminated source of energy that can be transformed into power without creating contamination and ecological harm. The photovoltaic materials and devices ease the translation of sunlight to electricity which is achieved through a photoelectric effect.^196^ Generally, the function of the device depends upon the material of which the device is manufactured. Therefore, various materials have been surveyed for device manufacture and characterization. GQDs as an exclusive class of 0D carbon nanomaterial have attracted marvellous attention in recent years due to its attractive physicochemical and electronic properties. Because of these unique properties, GQDs can enhance the efficiency of catalytic reactions in energy conversion applications. GQDs have been broadly scrutinized in photovoltaic technology due to their remarkable properties. The incorporation of GQDs into organic, inorganic and polymeric materials can improve electron movement while decreasing charge recombination and thus, increases the performance of solar cells. Before GQDs, graphene sheets were used in the photovoltaic devices for effective enhancement of power conversion efficiency (PCE). However, their direct application in nano-devices is restricted because the graphene sheet does not have an energy bandgap which can be explored by converting 2D graphene sheet into 0D quantum dots.^66,197^ Subsequently, with the invention of the photovoltaic devices, various types of solar converters have been manufactured and characterized to achieve high yield efficiency. A part of this review discusses the PCE of solar cell devices based on GQDs. Many researchers around the world have been investigating on several structures of solar cell devices based on generations which include quantum dot solar cells (QDSCs), dye sensitized solar cell (DSSC), polymer solar cells (PSCs), organic solar cells (OSCs), inorganic solar cells (IOSCs) and porphyrin sensitized solar cell (PSSC) which are focused to overcome the demand of electricity and improving PCE.

Generally, the efficiency of the solar cells is calculated by the absorption of incident photons and the amount of current generated. Table 5 summarizes the details of various solar cells and their performance based on factors such as open-circuit voltage (*V*_oc_), short-circuit current density (*J*_sc_), fill factor (FF), and PCE (*η*) that can be calculated from eqn (2)–(4) respectively.2
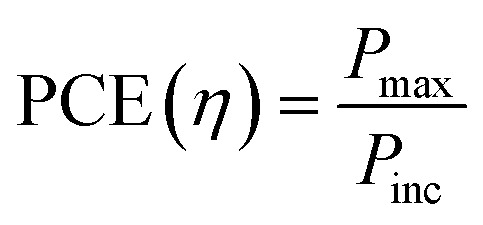
here *P*_inc_ is incident photon power and

3 *P*_max_ = FF × *V*_oc_ × *J*_sc_

where, FF is fill factor4
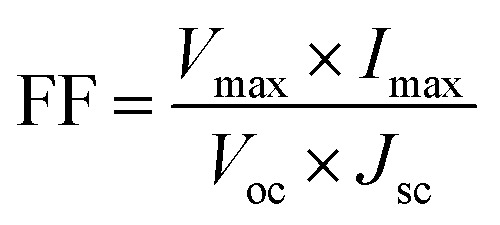
and *V*_oc_ & *J*_cc_ denote maximum open-circuit voltage and maximum close-circuit current respectively.^198^

**Table 5:** Summary of *J*–*V* characteristics of solar cells based on GQDs composites at different ratios

Type of solar cells	GQDs composites	*V*_oc_ (V)	*J*_sc_ (mA cm^−2^)	FF (%)	PCE (*η*) (%)	Ref.
Porphyrin sensitized solar cells (PSSCs)	GQD@ZnO/TCPPZn	0.48	10.1	50.7	2.45	198
PSCs	Few layered (F-GQDs) as HEL	0.75	15.20	69	7.91	199
PSCs	P3HT:PCBM/GQDs–CS_2_CO_3_ as cathode buffer layer	0.585	9.04	60	3.17	200
DSSCs	Ru based N719 acceptor dye	0.770	16.54	63	7.96	201
Perovskite solar cells (PSCs)	GQD/perovskite film	1.03 ± 0.03	23.60 ± 0.34	60.5 ± 2.2	14.7 ± 0.9	202
QDSCs	TiO_2_/CdS/GQDs as photoanode	0.740	—	12.34	5.96	203

Many investigators have discussed the role of GQDs and the effect of its concentration as a sensitizer material in different types of solar cells to achieve better PCE. Recently, Xu *et al.*^204^ fabricated PSC with different concentrations of ammonium iodide functionalized GQDs (GQDs-NI) as cathode interlayer material (CIL) with PCDTBT:PC71BM as the active layer and Ca as a CIL separately. The authors found good conductivity, high transparency, reduced charge recombination and improved charge extraction of GQDs-IN/PCDTBT:PC71BM because it has minimized the loss of photon and exploited the light absorption in the active layer. These results led to promising PCE which is about 7.49% *i.e.* much greater than that of the device with and without CIL (PCE = 6.72%) and (PCE = 5.38%) respectively. Additionally, Xie *et al.*^205^ investigated the PCE of PSCs based on perovskite SnO_2_ films with GQDs (SnO_2_:GQDs) as an electron-transporting layer. This composite material has achieved a maximum steady-state PCE of about >20.23% in comparison with the steady-state PCE of about 17.47% for bare SnO_2_ and also found the reduced photocurrent decay and improved photovoltage decay from 7.78 to 4.48 μs and 23.49 to 48.05 μs respectively. Hakimeh *et al.*^206^ have synthesized two types of GQDs namely GQD1 and GQD2 at two different temperatures for 8 and 48 h respectively through green synthesis method using corn powder as a starting material. It has been investigated as DSSCs which achieved 14.8% and 21.6% PCE while using GQDs as down conversion material. Gao *et al.*^207^ built up a solar cell device based on GQDs and achieved a conversion efficiency of about 6.63%. Such performance contrast with bare Si or GO can be clarified by the extraordinary energy band structure of GQDs, which act as an electron blocking layer for reducing the recombination at the anode.

**Fig. 19:**
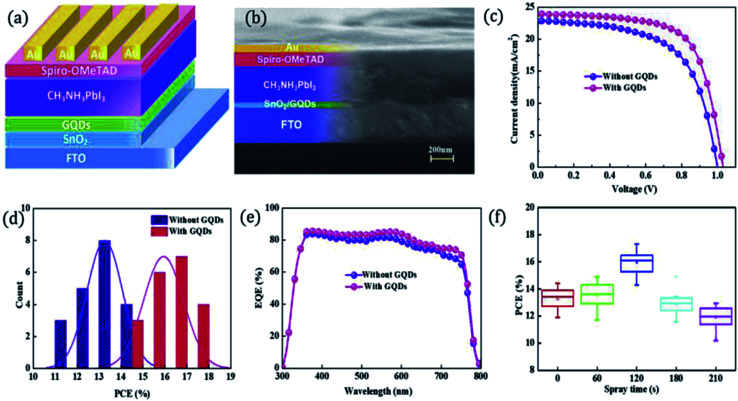
(a) schematic diagram of perovskite solar cell. (b) Cross sectional SEM image of perovskite structure. (c) *J*–*V* curves of the PSC based on SnO_2_ with and without GQDs. (d) Statistic histogram of the power conversion efficiency (PCE) (e) external quantum efficiency spectra. (f) PCE tendency with different time period. Reproduced with permission from ref. 211, copyright 2019, Elsevier.

**Fig. 20:**
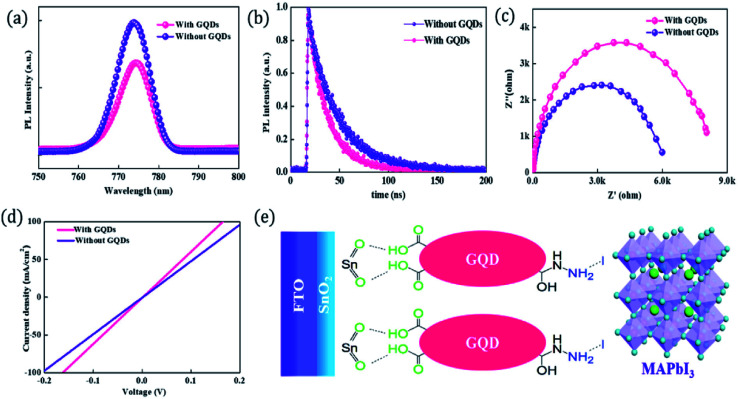
(a and b) Steady-state PL and TRPL spectrum of perovskite structure. (c) Electrochemical impedance spectrum of perovskite structure (d) *J*–*V* curves of the device under light environment. (e) Schematic diagram of the interfacial reaction between SnO_2_/GQDs and GQDs/perovskite. Reproduced with permission from ref. 211, copyright 2019, Elsevier.

Strikingly, the size of GQDs is expected to play a critical part in increasing the open-circuit voltage (*V*_oc_) of the device while short circuit current (*J*_sc_) lapses with the decrease of size. Due to quantum confinement effect of GQDs the heterojunction limit increases with the decreasing size while the impediment for gap transportation increases, realizing the development of *V*_oc_ and reduction of *J*_sc_, independently. The interfaces between materials, layers and different units are critical for the redesign of solar cells.^208–210^ For example, Xia *et al.* optimized the interfacial energy level alignment in perovskite solar cells and improved the *J*_sc_ and FF by modification with GQDs as showed in Fig. 19 and 20.^211^Fig. 19a shows the schematic diagram of the planar structure perovskite solar cell (PSC). Fig. 19b is the cross-sectional SEM image of the PSC device indicating that the functional layers are connected to each other which is advantageous to the charge transfer and enhancement of the resistance of the structure. Fig. 19c shows the *J*–*V* characteristics of the PSC based on SnO_2_ with and without GQDs. Comparison with bare SnO_2_, SnO_2_/GQDs has a little enhancement in *V*_oc_, *J*_sc_ and FF with values of 1.01 V, 22.32 mA cm^−2^ and 60.6%, respectively. Fig. 19d shows the statistical histogram of the PCEs with and without GQDs. PSC with GQDs have achieved higher PCE which is about 17.1%, than the device having no GQDs. The incident photon-to-current efficiency spectra of the device with and without GQDs electron transport layers, which is in good agreement with the *J*–*V* characteristics is shown in Fig. 19e. Fig. 19f box chat shows the effect of GQDs on the SnO_2_ layer by controlling the times for every 60 s (0–210 s). It indicates that the PCE increases as the time increases to 120 s. This also increases the values of *V*_oc_, *J*_sc_ and FF as well no significant improvement in PCE was observed when further increase the time from 120–210 s.

Fig. 20a and b shows the spectrum of the device based on SnO_2_/GQDs, which indicates faster emission quenching, more efficient electron extraction and transport with decreased average life time of charge carrier from 29.93 ns to 19.69 ns. Fig. 20c indicates that measurements of SnO_2_ and SnO_2_/GQDs in electrochemical impedance spectroscopy were carried out at 0.7 V bias voltage. The resultant diameter of the semicircle increased based on the SnO_2_/GQDs PSC device than bare SnO_2_. The interfacial contact of electron transport layers and perovskite layer was improved which obviously reduced the leakage and charge recombination at the interface. Fig. 20d demonstrates the conductivity of SnO_2_ and SnO_2_/GQDs composites measured by *J*–*V* curves under light conditions and Fig. 20e shows the schematic diagram of perovskite structure, which indicates attachment of functional groups at the edge GQDs and the interfacial contact of SnO_2_, respectively.^211^

#### 5.4.2 Dye sensitized solar cell

**Fig. 21:**
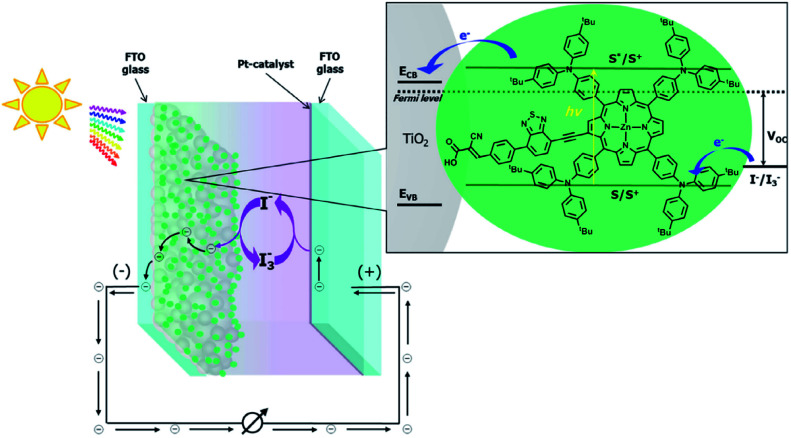
Working principle of the universal design of DSSC. Reproduced with permission from ref. 212, copyright 2018, Elsevier.

**Fig. 22:**
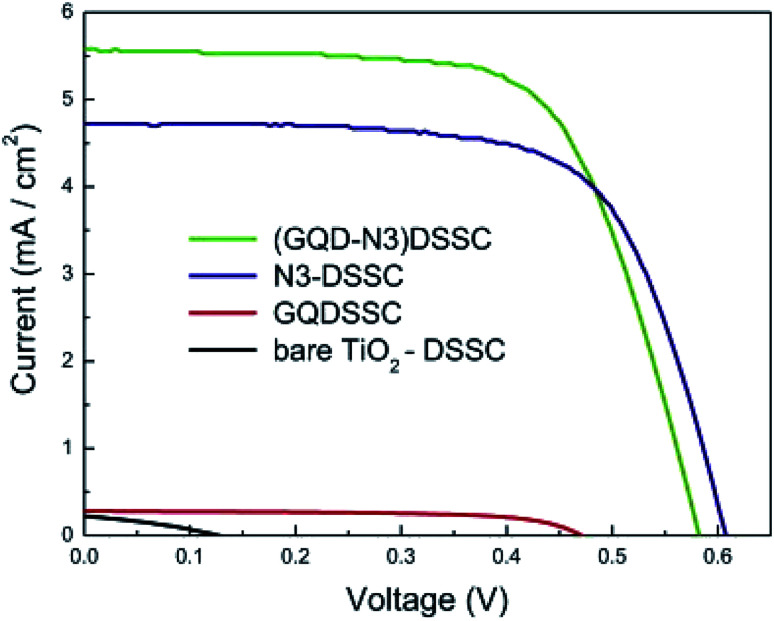
*J*–*V* characteristics curves of DSSCs with bare GQD, N_3_, TIO_2_ and combination of GQD and N_3_ were taken under 1 sun illumination intensity. Reproduced with permission from ref. 214, copyright 2015, Elsevier.

DSSC is a type of solar cell developed as an alternative for traditional solar cells being used in low-cost photovoltaic devices with attractive PCE. The universal structure of DSSC consists of a dye-sensitized photoanode (TiO_2_) and a platinum counter electrode with a liquid electrolyte redox mediator based on I_3_^−^/I^−^, Co^II^/Co^III^ or more recently on Cu^+^/2^+^ filling between cathode and anode as shown in Fig. 21.^212^ The fascinating properties of GQDs hold excellent results in various fields, especially in energy storage applications. In addition, the GQDs can produce various electrons by a single photon, while the dye cannot. In this manner, contrasted with the dye, the GQDs can produce more electrons to infuse into the TiO_2_, which would essentially upgrade the *J*_sc_ and *η* of the DSSCs. Subsequently, the GQDs play, somewhat, a similar role as the dye in DSSCs. Therefore, to make use of the outstanding properties of GQDs, which can turn into the DSSCs to improve the activity of the photoelectrode leading to enhancement of the efficiency of DSSCs. Here, GQDs are used as a co-sensitizer photoelectrode material along with TiO_2_.^212^ Fang *et al.*^213^ fabricated the DSSCs based on the GQDs adsorbed on the TiO_2_ and used as a photoelectrode material. The results demonstrated that by introducing the GQDs, the properties of the photoelectrode can be enriched significantly but the amount of dye used can be reduced. In another study, Mihalache *et al.*^214^ has prepared the cell based on the TiO_2_-GQDs composites as a sensitizer electrode material comprising of N_3_Ru dye, where an enhancement in PCE was attained. Fig. 22 shows that the *I*_sc_ and *V*_oc_ characteristics of GQD-N_3_ in DSSC which were higher than the bare TiO_2_.^214^

Liu *et al.*^215^ produced strontium ruthenate (SrRuO_3_) nanoparticles through the hydrothermal process and GQDs were decorated on the SrRuO_3_ to prepare SRO-GQDs hybrid. This hybrid counter electrode material was used in DSSC which achieved an impressive PCE of 8.05% which is much larger than the reference counter electrode (7.44%). Kundu *et al.*^216^ revealed an upgraded PCE of 11.7% ± 0.2 and a fill factor (FF) of 71% for DSSCs with a functioning area of 0.16 cm^2^ subsequent to altering the TiO_2_ photo-anode with size-specific (*ca.* 2 nm) N, F, S-codoped GQDs (NFS-GQDs). An upward shift in the Fermi level has been observed which may be responsible for the improved performance alongside the likelihood of avoiding the back electron move from TiO_2_. This work shows that the consolidation of size-controlled, hetero particle doped GQDs can upgrade the proficiency of DSSCs, empowering more optoelectronic applications. Table 6 summarized the *J*–*V* characteristics and PCE of DDSCs based on GQDs as a co-sensitizer material. Compared with other materials, the higher efficiency of DSSC which is 7.82% was achieved when GQDs were synthesized through the corn powder (green chemistry) and used as co-sensitized material.

**Table 6:** *J*–*V* characteristics and power conversion efficiencies of DSSCs based on GQDs

Type of cell	*V*_oc_ (V)	*J*_sc_ (mA cm^−2^)	FF	PCE (*η*) (%)	Ref.	
DSSCs based on GQDs	0.653 ± 0.02	22.62 ± 0.7	0.53 ± 0.06	7.82	206	
	0.66 ± 0.01	14.07 ± 0.02	0.59 ± 0.01	6.10 ± 0.01	213	
	0.583	5.58	0.66	2.15	215	

### 5.5 Catalytic applications

**Fig. 23:**
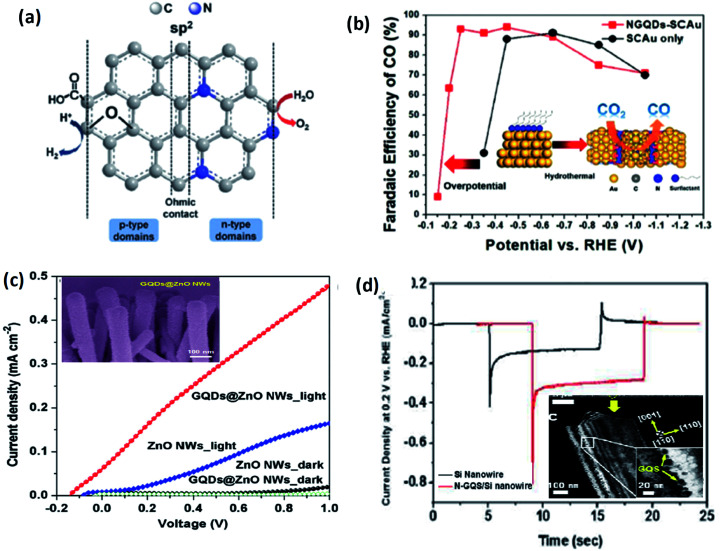
(a) Catalytic activity of GQDs. (The configuration of oxygen rich NGQDs domain). Reproduced with permission from ref. 176. Copyright 2014, Wiley Online Library. (b) Electrochemical conversion of CO_2_ to CO and faradic efficiency of NGQDs decorated gold nanoparticles. Reproduced with permission from ref. 223, copyright 2018, ACS Publications. (c) *I*–*V* of GQDs decorated ZnO nanowires PEC device. Inset shows the SEM image of GQDs decorated ZnO nanowires. Adapted from ref. 224, copyright 2013, Nature. (d) Transient curve of the photocurrent from device and inset shows the TEM image of GQDs decorated silicon nanowires. Reproduced with permission from ref. 226, copyright 2015, The Royal Society of Chemistry.

GQDs has strong catalytic properties and are widely used as a catalyst in processes like photocatalytic hydrogen evolution and CO_2_ reduction, electrocatalytic oxygen reduction, water splitting and CO_2_ reduction, as well as photoelectron catalysis. Photocatalysis needs proficient coupling of charge transfer and light-harvesting in the catalytic process. Various semiconductor metal oxide, employed for photocatalytic H_2_ production suffer from harnessing a broader range wavelength spectrum. Teng *et al.* revealed the intrinsic photocatalytic properties of GQDs in which GO sheets were used to synthesize NGQDs with nitrogen atoms embedded in the frame of GQD and oxygen groups functionalize the crystal surface (Fig. 23a).^176^ When NGQDs were combined with Pt as co-catalyst, the efficiency of 12.8% was observed for H_2_ production which is better than other metal-containing photocatalysts.^217^ On the other hand, when nitrogen and sulphur were co-doped with Pt-deposited GQDs, the H_2_ production efficiency was further improved to 29%.^218^ Another energy conversion process is electrocatalysis where chemical energy is directly converted to electrical power or *vice versa*. The important processes involved in this are the hydrogen evolution reaction (HER), oxygen evolution reaction (OER) and oxygen reduction reaction (ORR). A metal-free electrocatalyst of multi-walled carbon nanotubes decorated GQDs (GQDs/MWCNT) was reported by Qu *et al.*^219^ Compared to commercial Pt/C catalyst, the hybrid structure displayed enhanced electrocatalytic activities, strong methanol tolerance and long term stability. In another study, improved ORR onset potential of 0.07 V was demonstrated by incorporating C_3_N_4_ nanosheets and GQDs nanohybrid as a catalyst.^220^ An ORR onset potential closer to commercial Pt/C catalyst (0.01 V) was achieved by employing graphene coupled with nitrogen/boron co-doped GQDs as a catalyst.^221^ It is challenging to convert over-emitted CO_2_ into CO by electrochemical method. A substantially increased edge site defects produced by nitrogen doping in NGQDs demonstrated high catalytic activity towards CO_2_ electroreduction.^222^ The NGQDs oxygenates with high Faradaic efficiency (FE) up to 45% produces multi-carbon hydrocarbons which are comparable to copper-based electrocatalysts. Zhu *et al.* reported NGQDs decorated single-crystalline gold (Au) nanoparticles by converting the surfactant attached to the nanoparticle through the hydrothermal process (Fig. 23b).^223^ The synergetic effect of the NGQDs and gold nanoparticle significantly enhanced the conversion of CO_2_ to CO and a low onset potential of 0.15 V was achieved. In photoelectrochemical (PEC) cells GQDs served as alternative sensitizers to improve the light absorption ability of parent materials. A PEC photoelectrode with GQDs decorated ZnO nanowires, when used in water splitting showed prominent high short circuit current density and photo-induced open-circuit voltage compared to bare ZnO nanowire photoelectrode (Fig. 23c).^224^ Hong *et al.* incorporated NGQDs decorated p-type silicon nanowire as photocathode in solar driven HER.^225,226^ Compared to bare silicon nanowire applied bias photon-to-current efficiency of 0.91% the NGQDs modified device showed an efficiency of 2.29% and onset potential of 0.26 V which is much higher than that of other reported carbon-based PEC HER catalysts (Fig. 23d).^226^

## 6. Challenges and future perspectives

GQDs were proposed as a current-generation carbon material since their exceptional properties are suitable for many technological fields. However, there are some limitations that must be worked upon for its bright future. A complete understanding of the PL properties of GQDs is still worked upon and is awaited. Some potential mechanisms have been predicted such as the effect of size, modification of the surface, and doping with other elements. The methods drawn from the optical properties of the GQDs produced would be diverging, so complete learning of the basic working for PL in GQDs is very crucial. In the current scenario, GQDs have many hindrances, which includes the lack of proper synthesis technique that results in half estimation of optical characteristics, the lack of production in expected bulk sizes and morphology of GQDs without losing its optical properties, problems associated with PL emission wavelength and discrepancy on PL mechanism in a variety of applications. Also, in spite of the success of GQDs with unlike colour PL properties, with PL in the NIR state, the quantum yield of most GQDs has still not exceeded more than 55%. Thus, the up-gradation of GQDs is hindered because of their limited application in several areas rising from their poorer quantum yield. The bright potentials aspects of GQDs are strong, cost-effective, sophisticated and durable sensors that would work in any environmental conditions. The step-in synthesis of the GQDs should emphasis on addressing the present lacking in the production methods of GQDs to fulfill the wider application in various sensors, supercapacitors, solar cells, and biomedical devices. Additionally, the studies on GQDs associated with the applications in the field of ecological, bio-analysis and energy-related areas need to be conducted. Li-ion batteries are still at its early stage based on the GQDs as an electrode material. The GQDs hybrid with polymer matrix as an electrode material may satisfy many requirements of future power sources such as power densities and high energy, improve the cyclic stability of electrode, electrical conductivity, long lifetime, safety and environmental benignity, *etc.* It is significant to understand the emerging bare and functionalized GQDs in various aforementioned applications and to investigate the effect of their performance. This facet of the challenges provides more prospects for scientists working in the fields of material-based research and to strongly believe that GQDs can improve the higher expectations in future applications. The exploration of the physical and medical properties of GQDs are still in progress with natural substitutes. May be this would certainly be an eye-opening material to drug or gene delivery, biomedical, optical sensing and theranostic applications.

## 7. Conclusions

In this appraisal, the existing advancement of GQDs-based material used in various applications has been summarized. The material has been widely discussed and understood from the characteristics point of view. The studies concerning synthetic approaches, optical properties as well as present applications in which they are used have been pondered upon. Surprisingly, GQDs have exploded remarkable and increasing research interest especially in the field of sensors, because of their noticeable quantum confinement and edge effects, biocompatibility/nontoxicity and good chemical stability. PL, electrochemical, ECL, humidity sensor, bioimaging, supercapacitor and solar cells made of GQDs have been discussed in this review. A short review of morphologies of GQDs which is synthesized through different routes and their quantum yield has also been presented. However, the investigation on GQDs is still in the initial and prime platform. Most of the results presented in this review are related to GQDs which have been surfaced in the preceding three years. Nevertheless, GQDs based materials have shown many distinct advantages and great potential to replace some traditional materials.

## Conflicts of interest

All authors declare no conflict of interest.

## Supplementary Material
